# CD8^+^ T Cells Induce Fatal Brainstem Pathology during Cerebral Malaria via Luminal Antigen-Specific Engagement of Brain Vasculature

**DOI:** 10.1371/journal.ppat.1006022

**Published:** 2016-12-01

**Authors:** Phillip A. Swanson, Geoffrey T. Hart, Matthew V. Russo, Debasis Nayak, Takele Yazew, Mirna Peña, Shahid M. Khan, Chris J. Janse, Susan K. Pierce, Dorian B. McGavern

**Affiliations:** 1 Viral Immunology & Intravital Imaging Section, National Institute of Neurological Disorders and Stroke, National Institutes of Health, Bethesda, Maryland, United States of America; 2 Laboratory of Immunogenetics, National Institute of Allergy and Infectious Diseases, National Institutes of Health, Rockville, Maryland, United States of America; 3 Center for Bioscience and Biomedical Engineering, Indian Institute of Technology Indore, Madhya Pradesh, India; 4 Leiden Malaria Research Group, Department of Parasitology, Center of Infectious Diseases, Leiden University Medical Center, Leiden, The Netherlands; University of Pennsylvania, UNITED STATES

## Abstract

Cerebral malaria (CM) is a severe complication of *Plasmodium falciparum* infection that results in thousands of deaths each year, mostly in African children. The *in vivo* mechanisms underlying this fatal condition are not entirely understood. Using the animal model of experimental cerebral malaria (ECM), we sought mechanistic insights into the pathogenesis of CM. Fatal disease was associated with alterations in tight junction proteins, vascular breakdown in the meninges / parenchyma, edema, and ultimately neuronal cell death in the brainstem, which is consistent with cerebral herniation as a cause of death. At the peak of ECM, we revealed using intravital two-photon microscopy that myelomonocytic cells and parasite-specific CD8^+^ T cells associated primarily with the luminal surface of CNS blood vessels. Myelomonocytic cells participated in the removal of parasitized red blood cells (pRBCs) from cerebral blood vessels, but were not required for the disease. Interestingly, the majority of disease-inducing parasite-specific CD8^+^ T cells interacted with the lumen of brain vascular endothelial cells (ECs), where they were observed surveying, dividing, and arresting in a cognate peptide-MHC I dependent manner. These activities were critically dependent on IFN-γ, which was responsible for activating cerebrovascular ECs to upregulate adhesion and antigen-presenting molecules. Importantly, parasite-specific CD8^+^ T cell interactions with cerebral vessels were impaired in chimeric mice rendered unable to present EC antigens on MHC I, and these mice were in turn resistant to fatal brainstem pathology. Moreover, anti-adhesion molecule (LFA-1 / VLA-4) therapy prevented fatal disease by rapidly displacing luminal CD8^+^ T cells from cerebrovascular ECs without affecting extravascular T cells. These *in vivo* data demonstrate that parasite-specific CD8^+^ T cell-induced fatal vascular breakdown and subsequent neuronal death during ECM is associated with luminal, antigen-dependent interactions with cerebrovasculature.

## Author Summary

Cerebral malaria (CM) is a severe and potentially fatal complication of malaria in humans that results in swelling and bleeding within the brain. The mechanisms that cause this fatal disease in humans are not completely understood. We studied an animal model known as experimental cerebral malaria to learn more about the factors that drive this disease process. Using a technique referred to as intravital microscopy, we captured movies of immune cells operating in the living brain as the disease developed. At the peak of disease, we observed evidence of immune cells interacting with and aggregating along blood vessels throughout the brain. These interactions were directly associated vascular leakage. This caused the brain to swell, which gave rise to an unsustainable pressure that ultimately killed neurons responsible for heart and lung function. The fatal swelling was induced by immune cells (referred to as T cells) interacting with bits of parasite presented by blood vessels in the brain. Removal of this parasite presentation protected the mice from fatal disease. We also evaluated a straightforward therapy that involved intravenous administration of antibodies that interfered with T cell sticking to blood vessels. Our movies revealed that this therapeutic approach rapidly displaced T cells from the blood vessels in the brain and prevented fatal disease.

## Introduction

Malaria, a disease caused by protozoan parasites of the genus *Plasmodium*, is a leading cause of morbidity and mortality in the developing world. Of the 627,000 annual deaths due to malaria, the vast majority are caused by *Plasmodium falciparum* infections [1]. Human cerebral malaria (HCM) is one of several clinical manifestations of severe *P*. *falciparum* infection and is diagnosed by coma and parasitemia in the absence of meningitis, hyperglycemia, and postictal state [2]. HCM is fatal in 15–30% of affected individuals [3, 4], while an additional 10% of survivors suffer long-term neurological sequelae such as ataxia, hemiplegia, and cognitive impairment [5]. Yet, the underlying cause of HCM remains unknown.

Several characteristic pathologies are observed in the brains of patients suffering from HCM including vascular hemorrhage [6], breakdown of the blood brain barrier (BBB) [7, 8], and edema [9, 10]. At the cellular and molecular level, HCM is associated with an increase in systemic pro-inflammatory cytokines [11, 12], endothelial cell (EC) activation [13], and sequestration of parasite-infected red blood cells (iRBCs) [2] and leukocytes [8] within the brain vasculature. These conditions are hypothesized to contribute to the observed BBB disruption and cerebral edema as well as ischemia throughout the CNS [14]. However, interpretations of these HCM data are limited by the fact that most information about CNS pathology and the cellular response to *P*. *falciparum* is derived from post-mortem analyses. Although real-time *in vivo* imaging techniques such as MRI [9] and ophthalmoscopy [15] have been used in patients suffering from HCM, they lack the resolution needed to observe cellular dynamics in the CNS and have been used mostly to improve the fidelity of CM diagnoses. Examination of cellular dynamics in animal model systems is therefore needed to uncover mechanistic insights into HCM.

Infection of mice with *Plasmodium berghei* ANKA (PbA) induces a neurological disease called experimental cerebral malaria (ECM) that mirrors many of the pathological features observed in HCM. These include increased pro-inflammatory cytokines, vascular pathology, disruption of the BBB, and cerebral edema [16–19]. ECM in mice is a widely used model of HCM and provides a valuable tool for elucidating the mechanisms involved in CM pathogenesis and identifying cellular and molecular targets for adjunctive therapy [20]. Leukocytes have been shown to accumulate in the brains of mice throughout the course of ECM [21, 22]. In addition, mice that are genetically deficient in peripheral leukocyte chemokine receptors such as CCR5 and CXCR3 (or, their ligands) are resistant to ECM [23–26]. Many individual immune cell populations including neutrophils [27, 28], macrophages/monocytes [18, 29], NK cells [30], and CD4^+^ T cells [31, 32] have been implicated in the pathogenesis of this disease. However, other studies have shown that neither antibody-mediated nor genetic depletion of these cells affected the accumulation of iRBCs in the CNS [33] or the ability of mice to develop ECM [21, 34–36]. Therefore, the contribution of these immune cell subsets to ECM is still a matter of debate.

In contrast, the role of CD8^+^ T cells in ECM is unequivocal. Numerous studies have demonstrated that CD8^+^ T cell depletion [21, 31, 33, 34] or ablation of effector functions [22, 37] completely abrogates this disease. Furthermore, parasite-specific CD8^+^ T cells can mediate ECM in the absence of bystander T cells [38]. Despite the critical role played by CD8^+^ T cells during ECM, little is known about the dynamics, kinetics, anatomical localization, and function of these cells *in vivo*. One recent intravital study demonstrated that perivascular CD8^+^ T cell arrest is a signature of the disease [39], but it is unclear how these cells cause neurological symptoms or why mice succumb to ECM.

Using several techniques, including intravital imaging, we set out in this study to conduct an unbiased examination of how innate and adaptive immune cells contribute to cerebrovascular perturbations during ECM and to identify the cause of this fatal disease. We also sought *in vivo* insights into how cerebrovascular ECs respond to ECM and whether immune interactions with these cells could be therapeutically manipulated to ameliorate disease pathogenesis. Our data demonstrate that CD8^+^ T cells drive fatal vascular leakage during ECM, and they accomplish this by interacting in an antigen-dependent manner with cerebrovascular ECs. This results in profound BBB dysfunction and secondary death of neurons, most notably in the brainstem, which likely gives rise to autonomic dysfunction and death. ECM can be prevented by eliminating antigen presentation in cerebrovascular endothelial cells or by displacing parasite-specific CD8^+^ T cells from CNS blood vessels using an anti-adhesion molecule therapy.

## Results

### ECM is associated with recruitment of innate and adaptive immune cells to the CNS

**Fig 1:**
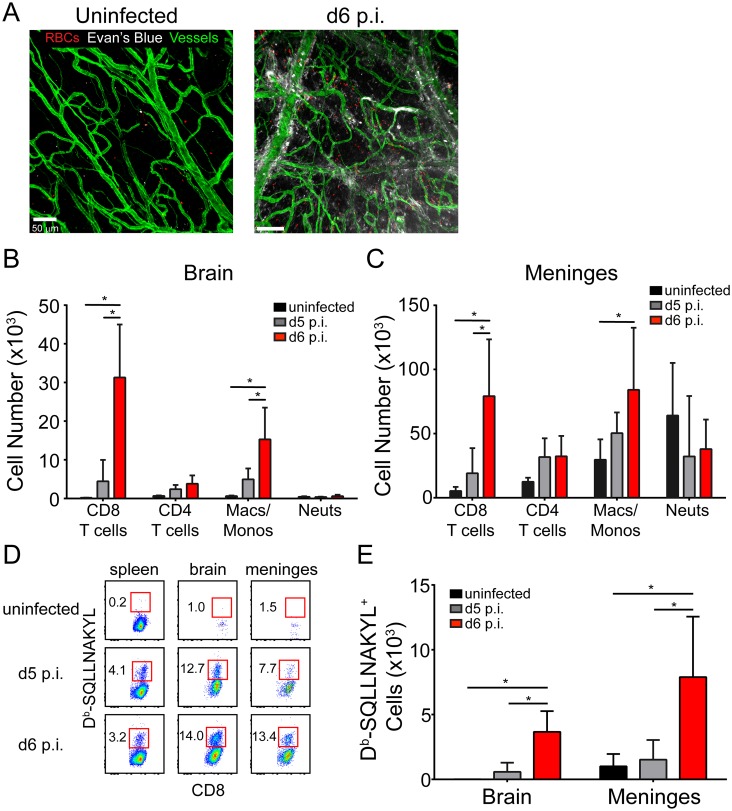
Meningeal pathology and kinetics of the CNS inflammatory response during ECM. (A) Representative confocal images of a meningeal whole mount from a naïve and d6 p.i. mouse following i.v. injection of Evans blue (white) (n = 4 mice per group). Evans blue and TER119^+^ red blood cells (red) leak from CD31^+^ vessels (green) into the meningeal space at d6 p.i. (B and C) Flow cytometric quantification of CD8^+^ T cells (CD45^+^Thy1.2^+^CD8^+^), CD4^+^ T cells (CD45^+^Thy1.2^+^CD8^-^), monocytes / macrophages (CD45^+^Thy1.2^-^CD11b^+^Ly6C^+^Ly6G^-^), and neutrophils (CD45^+^Thy1.2^-^CD11b^+^Ly6G^+^) in the brain (B) and meninges (C) of naïve, d5 p.i., and d6 p.i. mice. Bar graphs show mean ± SD (n = 5 mice per group). Data are representative of five independent experiments. (D) Representative flow cytometric dot plots show the frequency of D^b^-SQLLNAKYL-specific CD8^+^ T cells (red boxes and adjacent numbers) in the spleen, brain, and meninges naïve, d5 p.i., and d6 p.i. mice. Plots are gated on live CD45^+^ Thy1.2^+^ CD8^+^ cells. (E) Quantification of the data shown in (D). Bar graphs show mean ± SD (n = 5 mice per group). Data are representative of two independent experiments. Asterisks denote statistical significance (*P < 0.05).

Human cerebral malaria is associated with several hallmark pathologies in the brain parenchyma including vascular hemorrhaging, breakdown of the BBB, and cerebral edema. Similarly, we observed that mice infected with PbA were highly parasitemic, moribund, and showed evidence of profound BBB breakdown and edema at day 6 p.i. (S1A–S1E Fig). We also observed evidence of significant vascular hemorrhaging in the brain parenchyma (S1F Fig) that was almost always associated with iRBCs (S1G Fig). Interestingly, evidence of vascular pathology was also found in the meninges, which has not been reported previously (Fig 1A).

To gain insights into the immunopathogenesis of ECM, we conducted a temporal analysis of the immune subsets that arrive in the CNS as PbA-infected mice develop neurological symptoms. We used a clinical scoring system of 0 (moribund) to 20 (asymptomatic) developed by Carroll *et al*. [40] to assess disease severity (S1A Fig). We separately evaluated the meninges and brains of naïve, d5 p.i. (parasitemic but asymptomatic), and d6 p.i. (highly parasitemic and symptomatic) mice. This study revealed a significant increase in CD8^+^ T cells and Ly6C^hi^ inflammatory macrophages/monocytes in the brains and meninges as mice developed symptoms on d6 p.i. (Fig 1B and 1C). To monitor PbA-specific CD8^+^ T cells, we generated pentamers consisting of H-2D^b^ loaded with an immunodominant PbA epitope (SQLLNAKYL) described by Howland *et al*. [41]. Interestingly, PbA-specific CD8^+^ T cells increased in frequency and number in the spleen, meninges, and brain as mice progressed from asymptomatic (d5 p.i.) to symptomatic (d6 p.i.) (Fig 1D and 1E). Thus, neurological symptoms during ECM are associated with the migration of Ly6C^hi^ monocytes and parasite-specific CD8^+^ T cells to the CNS.

### Innate immune cells accumulate within cerebral vasculature but do not cause fatal disease

**Fig 2:**
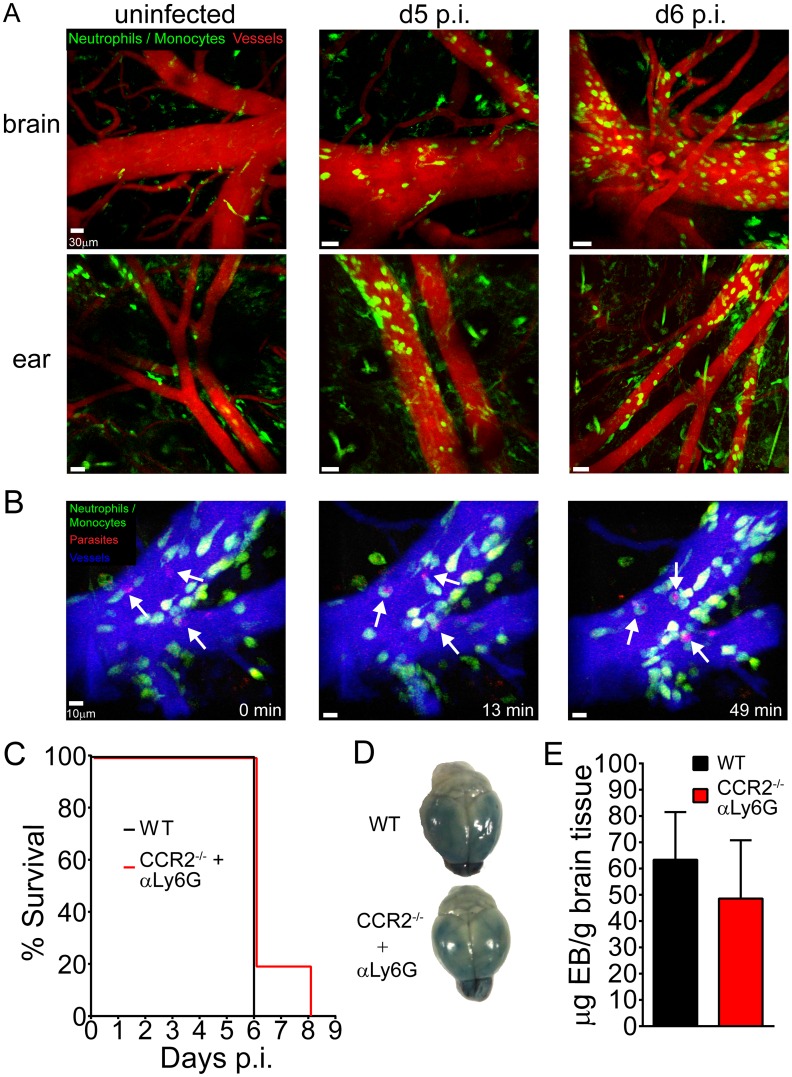
*In vivo* myelomonocytic cell dynamics and their contribution to ECM-mediated pathology. (A) Representative maximal projections of 3D time lapses were obtained from the thinned skull window (top) and corresponding ear (bottom) of naïve, d5 p.i., and d6 p.i. LysM^gfp/+^ mice. Note the accumulation of myelomonocytic cells (green) along brain and ear vessels (red) visualized with quantum dots (n = 3–4 mice per group). See corresponding S1 Movie. (B) A representative 3D time lapse of the brain was obtained from a LysM^gfp/+^ mouse infected with PbA-OVA-mCherry at d6 p.i. Myelomonocytic cells (green) were observed phagocytosing parasites (red) attached to the lumen of cerebral blood vessels (blue) (denoted with white arrows). Time points in the image series are denoted in minutes. See corresponding S3 Movie. (C) Survival curve for PbA-infected wild type mice (black line) and CCR2^-/-^ mice that were depleted of neutrophils following i.p. injection of anti-Ly6G at day -1 (red line) (n = 4–5 mice per group). (D) Representative brains from wild type (top) and CCR2^-/-^ (bottom) mice depleted of neutrophils at d-1. Mice were injected i.v. with Evans Blue dye at d6 p.i. (E) Fluorometric quantification of data shown in (D). Data are represented as mean ± SD (n = 5 mice per group). All data in this figure are representative of two independent experiments. Asterisks denote statistical significance (*P < 0.05).

Because myeloid cells are recruited to the CNS during ECM, we examined the dynamics and anatomical distribution of these cells using intravital two-photon microscopy (TPM) through a thinned skull window as described [42, 43]. We monitored the dynamics of myelomonocytic cells (monocytes and neutrophils) using lysozyme M-GFP (LysM^gfp/+^) reporter mice [44]. Consistent with our flow cytometric data Fig 1B and 1C we observed a significant increase in the number of myelomonocytic cells in symptomatic mice at d6 p.i. compared to d5 p.i. and uninfected animals. Interestingly, the myelomonocytic cells were contained almost entirely within the vasculature and were found in both the brain and periphery (ear) indicating systemic inflammation (Fig 2A) (S1–S3 Movies). These cells were also occasionally associated with vascular leakage in the brain (S2 Movie).

To determine the anatomical relationship between myelomonocytic cells and iRBCs during the development of ECM, we infected LysM^gfp/+^ mice with recombinant PbA expressing mCherry and OVA (PbA-OVA-mCherry) [45]. In symptomatic mice at d6 p.i., we observed fluorescent iRBCs adherent to cerebral vasculature (Fig 2B; S3 Movie), similar to what is found in human CM patients. We also visualized myelomonocytic cells actively phagocytosing these iRBCs while patrolling the vascular lumen (Fig 2B; S3 Movie). In fact, we found that on average nearly 60% of the fluorescent iRBCs were associated with LysM-GFP signal at any one time. In addition to the vascular lumen, some fluorescent iRBCs localized to the perivascular spaces of intact vessels, suggesting extravasation (S4 Movie). These parasites were rapidly acquired by perivascular macrophages visualized in CX3CR1^gfp/+^ mice (S4 Movie). These data indicate iRBCs were positioned on the luminal and abluminal surface of cerebral blood vessels during the development of ECM.

The contribution of innate immune cells to the pathogenesis of ECM is still a matter of contention. Previous studies have either implicated monocytes and neutrophils in the pathogenesis of ECM [18, 27–29] or shown them to be irrelevant [21, 33, 35, 36]. Our flow cytometric (Fig 1) and imaging (Fig 2) data suggested that these cells might be involved in disease pathogenesis. To determine if myelomonocytic cells contribute to BBB breakdown or mortality during ECM, we depleted neutrophils with anti-Ly6G antibodies in CCR2^-/-^ mice (S2A Fig, which are deficient in circulating monocytes [46, 47]. This resulted in an 85% reduction of the total circulating myelomonocytic compartment. When compared to PbA-infected controls, mice lacking monocytes and neutrophils showed no preservation of BBB integrity or reduced mortality rate during ECM (Fig 2C–2E). In addition, depletion of myelomonocytic cells did not improve clinical scores or alter parasitemia levels (S2B and S2C Fig). Based on these results, myelomonocytic cells do not appear to play a significant role in the pathogenesis of ECM.

### CD8^+^ T cells arrest along the CNS vasculature and cause ECM

**Fig 3:**
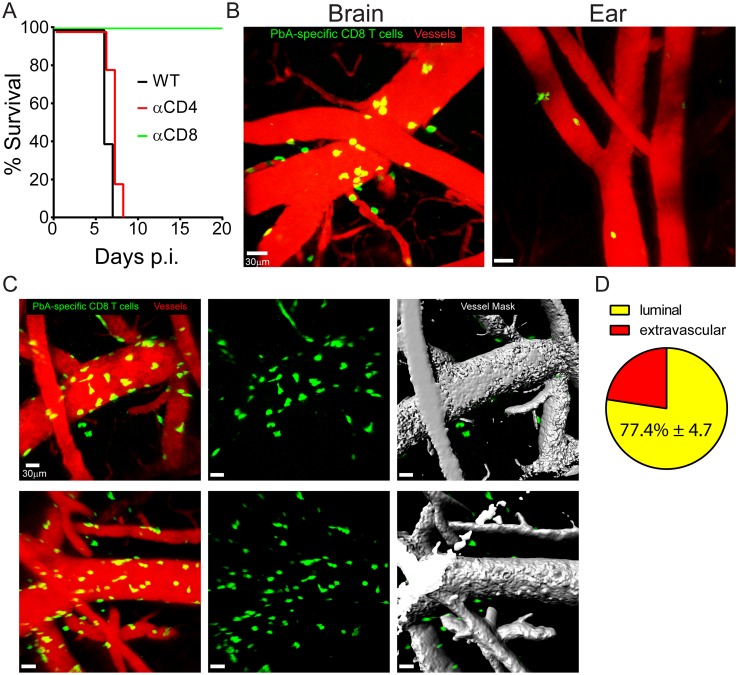
CD8^+^ T cells localize preferentially to cerebral vasculature and promote fatal vascular breakdown during ECM. (A) Survival curve showing PbA-infected wild type (black line), CD4^+^ T cell depleted (red line), and CD8^+^ T cell depleted (green line) mice over time (n = 4–5 mice per group). CD8^+^ or CD4^+^ T cells were depleted by injecting anti-CD8 or CD4 antibodies, respectively, at d4 p.i. (B) Representative maximal projections of 3D time lapses captured through a thinned skull window and the corresponding ear of a wild type mouse seeded i.v. with 10^4^ naïve mCerulean^+^ OT-I cells (green) and then infected one day later PbA-OVA-mCherry. Time lapses were captured at d6 p.i. Blood vessels are shown red (n = 7 mice per group). See corresponding S5–S7 Movies. (C) Maximal projections captured through a thinned skull window depicting OT-I cells (green), blood vessels (red), and a volumetric mask corresponding to the vascular quantum dot signal (gray). Each row represents a different mouse. See corresponding S8 Movie. (D) Pie graph showing the percentage of luminal vs. extravascular OT-I cells at day 6 p.i. (mean ±SD; n = 5 mice). All data in this figure are representative of two independent experiments. Asterisks denote statistical significance (*P < 0.05).

Having ruled out myelomonocytic cells as the cause of fatal pathology during ECM, we focused on the adaptive immune response, with a specific emphasis on T cells given that mice lacking B cells are still susceptible to ECM [34]. We initiated this line investigation by conducting a series a T cell depletion experiments. On d4 p.i. PbA-infected mice were administered antibodies specific for CD8^+^ or CD4^+^ T cells (S3A Fig) and then monitored for development of ECM relative to untreated control mice (Fig 3A). Although depletion of CD4^+^ T cells had no effect on survival, CD8^+^ T cell depleted mice were completely resistant to ECM (Fig 3A) despite having levels of parasitemia comparable to control mice (S3B Fig). Depletion of CD8^+^ T cells also prevented the vascular hemorrhaging (S3C Fig), BBB breakdown (S3D and S3E Fig), and edema (S3F Fig) normally associated with ECM.

To determine the anatomical localization of activated PbA-specific CD8^+^ T cells within the brain at the peak of ECM, naïve B6 mice were seeded i.v. with 10^4^ naïve mCerulean^+^ OT-I T cell receptor transgenic CD8^+^ T cells and then infected with PbA-OVA-mCherry. Symptomatic mice were imaged 6 days later by TPM. These intravital imaging studies revealed that nearly all PbA-specific CD8^+^ T cells in symptomatic mice were arrested on or slowly crawling along the luminal and extravascular surfaces of cerebral blood vessels and were often associated with significant vascular breakdown. (Fig 3B; S5–S7 Movies). Interestingly, this activity was specific to the brain, as PbA- specific CD8^+^ T cells were not observed arresting along the vasculature in a peripheral tissue (ear) within the same mouse (Fig 3B; S5 Movie). Although PbA-specific CD8 T cells in the CNS were observed within perivascular spaces and the parenchyma, the majority appeared to be interacting with the luminal surface of blood vessels. To quantify the percentage of luminal vs. extravascular PbA-specific CD8 T cells, we created volumetric masks corresponding to the Evans blue signal in each blood vessel (Fig 3C, S8 Movie). After applying the mask, all visible PbA-specific CD8^+^ T cells, including those located on the surface blood vessels, were counted as extravascular, whereas cells obscured by the mask were considered luminal (Fig 3C, S8 Movie). We found that the vast majority of PbA-specific CD8 T cells were associated with the luminal surface of CNS blood vessels (Fig 3D). Collectively, these data indicate that CD8^+^ T cells arrest along the cerebral vasculature during ECM and are responsible for the vascular pathology.

### Fatal ECM is associated with severe brainstem pathology

**Fig 4:**
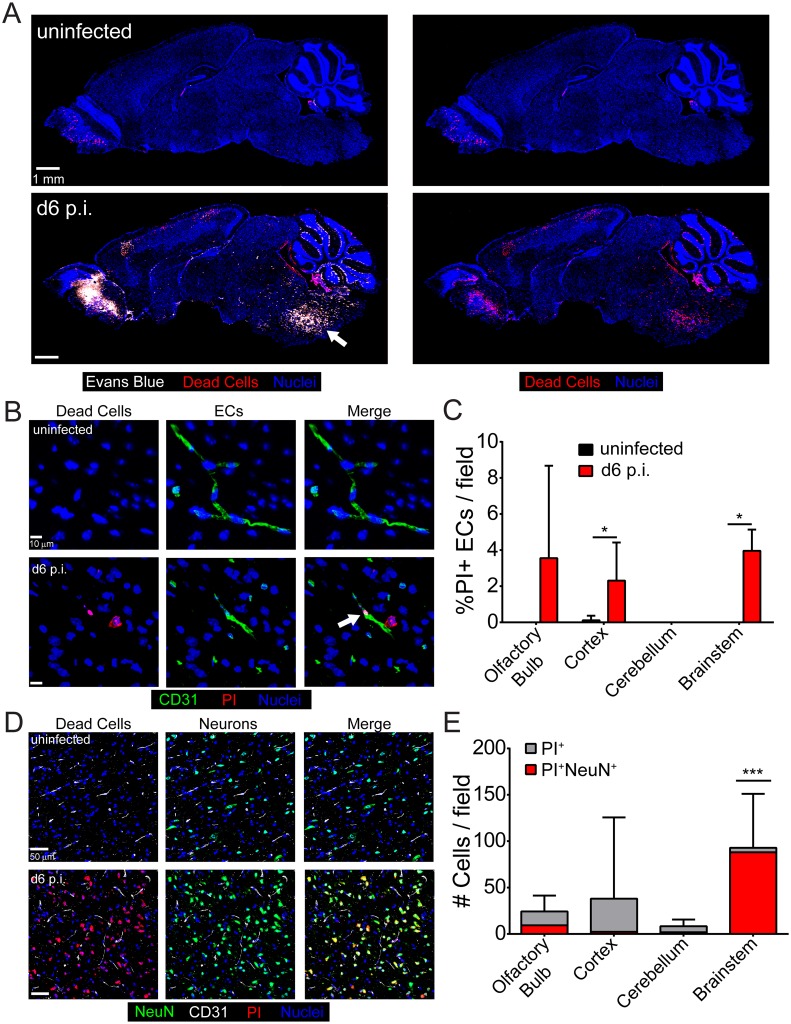
Vascular leakage and severe brainstem pathology during ECM. (A) Representative confocal images of sagittal brain sections from an uninfected mouse (top) or a symptomatic mouse at d6 p.i. following simultaneous i.v. injection of Evans blue (white) and propidium iodide (red) to visualize vascular leakage and cell death, respectively (n = 5 mice per group). Cell nuclei are shown in blue. Note the presence of severe brainstem pathology (white arrow) in the d6 p.i. mouse. (B) Representative confocal images of brain sections from a naïve (top) or a symptomatic mouse at d6 p.i. following propidium iodide (red) injection and immunohistochemical staining for CD31^+^ ECs (green). Cell nuclei are shown in blue. (C) The bar graph depicts quantification of data shown in (B). Data are represented as the percentage of PI^+^ CD31^+^ ECs of total CD31^+^ ECs per brain region (mean ± SD; n = 5 mice per group; 1 brain section per mouse; 10 images per brain section; 2–3 images per brain region). Asterisks denote statistical significance (*P < 0.05). (D) Representative confocal images of brain sections from an uninfected (top) or a symptomatic mouse at d6 p.i. following propidium iodide (red) injection and immunohistochemical staining for NeuN^+^ neurons (green). Cell nuclei are shown in blue. (E) The bar graph shows quantification of data shown in (D). Each bar represents the total number of PI^+^ cells in each brain region (gray), and within the bar, the red coloration shows the proportion that are also NeuN^+^ (mean ± SD; n = 4 mice per group; 1 brain section per mouse; 12 images per brain section; 3 images per brain region). Asterisks denote a statistically significant difference from all other groups (***P < 0.05).

Because PbA-specific CD8^+^ T cells were intimately associated with cerebral vasculature, we postulated that cytotoxic lymphocyte-mediated killing of vascular ECs might serve as the cause of BBB breakdown and death during ECM. To simultaneously assess cell death and vascular leakage *in vivo*, we injected naïve and symptomatic mice at d6 p.i. intravenously with propidium iodide (PI) (to label dead cells) and Evans blue (to assess vascular leakage). This assay revealed a striking pattern of pathology in all mice succumbing to ECM (Fig 4A). Whereas evidence of cell death was observed in multiple brain regions (e.g. olfactory bulb, cortex, cerebellum, brainstem, choroid plexus), the brainstem and olfactory bulb pathology were particularly severe (Fig 4A). Both brain regions showed evidence of profound vascular leakage and cell death. The brainstem pathology is consistent with cerebral herniation [48] and would likely give rise to autonomic dysfunction. To determine if the PI^+^ cells were in fact vascular ECs, we co-stained sagittal brain sections with anti-CD31 antibodies and performed quantitative analyses (Fig 4B and 4C). Although EC death was observed in multiple brain regions during ECM, only a small fraction of the total ECs was PI^+^ (Fig 4B and 4C). Thus, it is unlikely that EC death is the cause of fatal disease in mice with ECM.

We found a small number of PI^+^ ECs in the brain during ECM, but the vast majority of the dead cells were unknown. Based on cellular morphology and anatomical location, we hypothesized that at least some of the PI^+^ cells were neurons. Interestingly, co-staining with anti-NeuN antibodies revealed that nearly all of the PI^+^ cells in the brainstem were neurons—a pattern of cell death that was unique to this brain region (Fig 4D and 4E). Because the brainstem controls vital functions such as the cardiovascular and respiratory systems, it is likely that mice succumb to ECM due to the widespread neuronal death observed in this brain region.

### Tight junction protein expression is reduced only in areas of vascular breakdown

**Fig 5:**
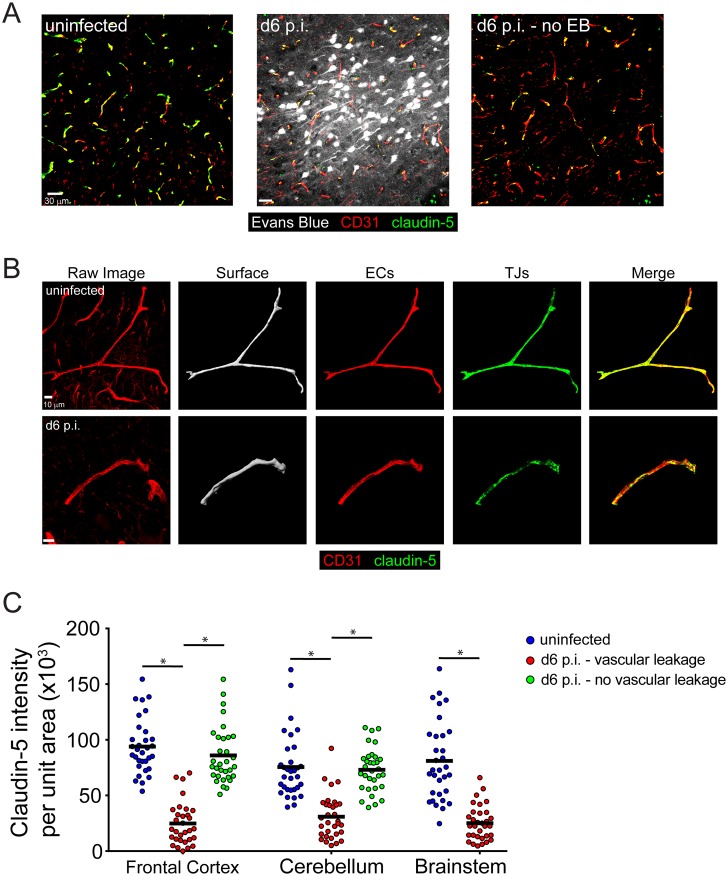
TJ protein expression is reduced in areas of vascular breakdown. (A) Representative confocal images from the frontal cortex of an uninfected mouse (left) or a symptomatic mouse at d6 p.i. (middle, right) following i.v. injection of Evans blue dye (white) to visualize vascular leakage. (n = 4 mice per group). (B) Representative confocal images of cerebral blood vessels from an uninfected mouse (top) or a symptomatic mouse at d6 p.i. (bottom) following immunohistochemical staining for CD31^+^ ECs (red) and the TJ protein claudin-5 (green). The volumetric mask of the blood vessel generated from the CD31 signal is shown in the second panel. (C) The bar graph shows quantification of the data in (B) plus areas of no vascular hemorrhage from the same symptomatic mice at d6 p.i. Each symbol represents one blood vessel. (n = 4 mice per group, 1 section per mouse, 3–4 images per brain region). Asterisks denote statistical significance (*P<0.05).

We observed evidence of profound vascular leakage in several brain regions, including the brainstem; however, previous studies have suggested that this is not due to reduced expression of endothelial tight junction proteins [18]. We hypothesized that this negative result might be explained by a failure to directly compare tight junction (TJ) protein expression in leaking versus intact cerebral vasculature during the development of ECM. To test this hypothesis, we injected uninfected or symptomatic mice at d6 p.i. with Evans blue to locate areas of vascular leakage within the brain (Fig 5A). Next, we stained sagittal brain sections with antibodies against CD31 and claudin-5 to identify ECs and TJs, respectively (Fig 5B). Thick sections were used in order to generate volumetric 3D masks of individual blood vessels in the frontal cortex, cerebellum, and brainstem (Fig 5B). This method provides a more accurate representation of claudin-5 staining over an entire blood vessel than would be obtained by performing 2D analyses on thin sections. When we quantified the intensity of claudin-5 staining on 3D reconstructed blood vessels in various regions of the brain, we consistently found reduced expression in areas of Evans blue+ vascular leakage within symptomatic mice when compared to the same areas in uninfected mice (Fig 5C). Furthermore, we found that claudin-5 levels in brain regions of symptomatic mice where there was no vascular leakage were comparable to the levels observed on naïve blood vessels (Fig 5C). We were unable to find areas without vascular leakage in the brainstem due to the extensive amount of pathology in this brain region. Thus, by comparing to leaking to intact cerebral blood vessels, we uncovered that vascular leakage is indeed associated with reduced TJ expression.

### CNS ECs are selectively activated in an IFN-γ-dependent manner during ECM

**Fig 6:**
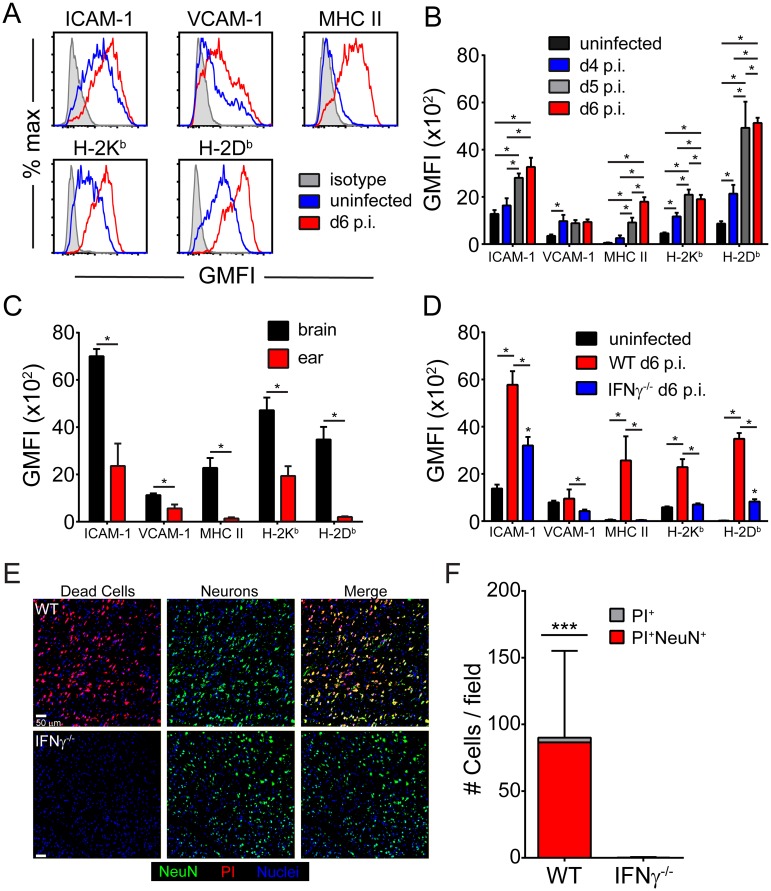
Cerebral ECs upregulate antigen presenting and adhesion molecules in an IFNγ-dependent manner. (A) Representative histograms from naïve (blue) and d6 p.i. (red) mice show the expression antigen presenting (D^b^, K^b^, MHC II) and adhesion molecules (ICAM-1, VCAM-1) on live CD45^-^ CD31^+^ ECs. Isotype control antibody staining is shown in gray. (B) The bar graph depicts the geometric mean fluorescent intensity of the denoted molecules on cerebral ECs extracted from naïve, d4 p.i., d5 p.i., and d6 p.i. mice (mean ± SD; n = 5 mice per group). (C) The bar graphs shows expression of the same molecules on ECs extracted from the brain vs. ear of mice at d6 p.i. (mean ± SD; n = 5 mice per group). (D) Expression of antigen-presenting and adhesion molecules on cerebral ECs were quantified for naïve, wild type d6 p.i., and IFNγ^-/-^ d6 p.i mice (mean ± SD; n = 5 mice per group). Note that IFNγ deficiency restores expression to near baseline levels. Data in this figure are representative of two independent experiments. (E) Representative confocal images of the brainstem from a symptomatic wild type mouse (top) or an IFNγ^-/-^ mouse at d6 p.i. following propidium iodide (red) injection and immunohistochemical staining for NeuN^+^ neurons (green). Cell nuclei are shown in blue. (F) The bar graph shows quantification of data in (E). Each bar represents the total number of brainstem cells that are PI^+^ (gray), and within the bar, the red coloration shows the proportion that are also NeuN^+^ (mean ± SD; n = 4 mice per group; 1 brain section per mouse; 4 images per brain section). Asterisks denote a statistically significant difference from all other groups (***P < 0.05).

We observed the PbA-specific CD8^+^ T cells interact heavily with cerebral vasculature during ECM, but the vast majority of ECs survive these engagements. To better understand the mechanisms guiding these interactions, we conducted flow cytometric analyses of cerebral ECs during the development of ECM and compared them to ECs extracted from a peripheral tissue (ear) (Fig 6A–6C). By gating on live, CD45^-^CD31^+^ cells (S4A Fig), we noted that adhesion (ICAM-1, VCAM-1) and antigen-presenting (I-A^b^, D^b^, K^b^) molecules were all significantly increased on ECs from symptomatic mice at d6 p.i. (Fig 6A and 6B). Elevated expression of these molecules appeared as early as d4 p.i. and usually increased further as the disease progressed (Fig 6B). ECs extracted from meningeal blood vessels were similarly activated (S4B Fig). Interestingly, this EC activation phenotype was unique to the CNS, as ECs extracted from a peripheral site (ear) showed a significantly reduced expression level of adhesion and antigen presentation molecules (Fig 6C). This is consistent with our intravital imaging data showing slow crawling and arrest of PbA-specific CD8^+^ T cells along cerebral, but not ear vasculature (Fig 3B; S5 Movie). This finding also suggests that the increase in myelomonocytic cells observed in the ear vasculature of PbA-infected mice did not induce EC activation.

We next sought insights into the mechanism underlying cerebral EC activation during ECM. Previous studies have shown that IFNγ-deficient mice are protected from ECM [34, 49, 50]. We confirmed these findings (S4C–S4E Fig) and hypothesized that IFNγ produced by lymphocytes recruited to the CNS might be responsible for cerebral endothelial cell activation during ECM (Fig 6B). A link has been established between IFNγ and ICAM-1 expression in the brain during ECM, but the cell type(s) affected by the absence of IFNγ was not determined [51, 52]. To address the role of IFNγ in EC activation during ECM, we infected wild type and IFNγ^-/-^ mice with PbA and examined EC phenotype on day 6. ECs from IFNγ^-/-^ mice had significantly reduced levels of adhesion and antigen presentation molecules (Fig 6D). In fact, with the exception of ICAM-1 and H-2D^b^, the loss of IFNγ reduced the activation state of brain ECs to uninfected levels. These data correlated with a severe reduction in PbA-specific CD8 T cell accumulation in the brain, despite normal peripheral expansion and migration (S4F Fig). Furthermore, PbA-infected IFNγ^-/-^ mice lacked the severe vascular breakdown and brainstem neuronal cell death observed in wild type mice (Fig 6E and 6F). These data indicate that IFNγ is responsible for cerebral EC activation during ECM and that IFNγ-deficiency likely protects mice in part by keeping ECs in a naïve state.

### Late blockade of adhesion molecules disrupts PbA-specific CD8^+^ T cell-EC interactions and protects mice from ECM

Previous studies using CBA/J mice have demonstrated that antibody blockade of LFA-1 during ECM is highly efficacious at preventing disease [17, 53, 54]. However, we have found that this treatment is ineffective in PbA-infected C57BL/6 mice. Activated CD8^+^ T cells express multiple adhesion molecules, including LFA-1 and VLA-4, which are ligands for ICAM-1 and VCAM-1, respectively. Given that brain ECs upregulate both ICAM-1 and VCAM-1 during ECM (Fig 6B), we hypothesized that administration of a combination of anti-LFA-1 and anti-VLA-4 antibodies (anti-LFA-1/VLA-4) could be used to therapeutically displace PbA-specific CD8^+^ T cells from cerebral vasculature and prevent fatal disease. We administered this therapy on day 5.5 post-infection to avoid interfering with T cell priming. Mice at this time point were parasitemic (S5B Fig) and had symptoms associated with ECM (S5C Fig). Importantly, treatment with anti-LFA-1/VLA-4 completely reversed these symptoms and prevented death in PbA-infected mice (S5A and S5C Fig). This treatment had no effect on PbA-specific CD8^+^ T cell expansion (S5D and S5E Fig).

**Fig 7:**
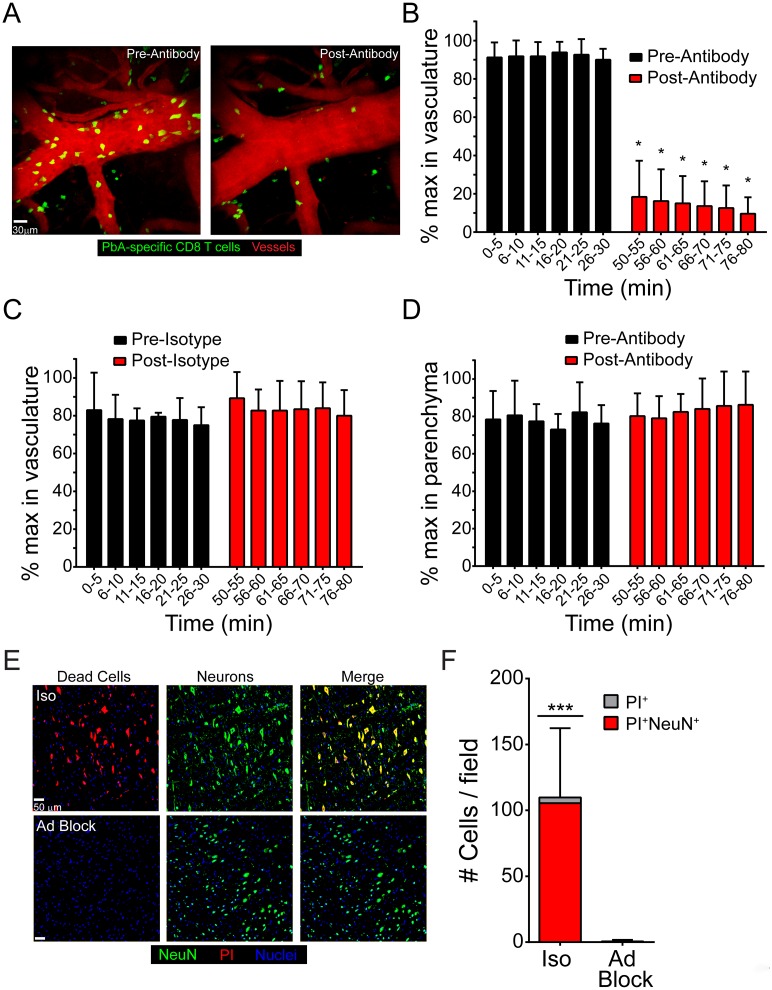
Adhesion molecule blockade displaces PbA-specific CD8^+^ T cell from cerebral blood vessels and promotes survival. (A) Representative maximal projections of 3D time lapses captured through the thinned skull window of a mouse seeded i.v. with 10^4^ naïve mCerulean^+^ OT-I cells (green) and then infected with PbA-OVA-mCherry. Images were captured at d6 p.i. The left panel shows the distribution of OT-I cells along cerebral blood vessels (red) before antibody blockade and the right panel depicts the same vessel after treatment. See corresponding S9 Movie. (B and C) The bar graphs show quantification of luminal PbA-specific CD8^+^ T cells as a percentage of maximum number in brain vasculature at the denoted time points before and after i.v. treatment with anti-LFA-1 / VLA-4 (B) or isotype control antibodies (C). Data are representative of 4–5 independent experiments. (D) Quantification of PbA-specific CD8^+^ T cells in the brain parenchyma as a percentage of the maximum number after i.v. treatment with anti-LFA-1/VLA-4 antibodies. Data are representative of 5 independent experiments. (E) Representative confocal images of the brainstem from mice at d6 p.i. following propidium iodide (red) injection and immunohistochemical staining for NeuN^+^ neurons (green). Mice were treated with either isotype control antibody (top) or anti-LFA-1/VLA-4 blocking antibodies (Ad block) at d5.5 p.i. Cell nuclei are shown in blue. (F) The bar graph shows quantification of data shown in (E). Each bar represents the total number of brainstem cells that are PI^+^ (gray), and within the bar, the red coloration shows the proportion that are also NeuN^+^ (mean ± SD; n = 4 mice per group; 1 brain section per mouse; 4 images per brain section). Asterisks denote a statistically significant difference from all other groups (***P < 0.05).

To assess the impact of anti-LFA-1/VLA-4 therapy on PbA-specific CD8^+^ T cell dynamics in the brain, we performed a series intravital imaging studies. At day 6 following infection with PbA-OVA, we imaged the dynamics of mCerulean^+^ OT-I T cells in the brain for 30 min by TPM. This was followed by intravenous administration of anti-LFA-1/VLA-4 therapy and an additional 30 min of imaging in the same anatomical location. Before antibody treatment, PbA-specific CD8^+^ T cells were observed slowly crawling along and arresting on cerebral blood vessels (Fig 7A; S9 Movie). In contrast, anti-adhesion antibody treatment resulted in an immediate displacement of PbA-specific CD8^+^ T cells from the vasculature (Fig 7A; S9 Movie). A significant reduction in the frequency of PbA-specific CD8^+^ T cells associated with brain vasculature was observed for the entire viewing period after anti-adhesion therapy (Fig 7B), which was not seen in mice treated with istoype control antibodies (Fig 7C). Interestingly, the frequency of PbA-specific CD8^+^ T cells in the parenchyma and perivascular spaces was not affected by blocking adhesion molecules (Fig 7D), suggesting that intravascular CD8^+^ T cell interactions are the ones responsible for fatal pathology during ECM. Furthermore, disruption of luminal CD8 T cell interactions with brain ECs via adhesion molecule blockade also prevented the death of brainstem neurons observed in isotype control treated mice (Fig 7E and 7F). In concert, these data show that blocking adhesion to ICAM-1 and VCAM-1, which are highly expressed on brain ECs during ECM, prevents PbA-specific CD8^+^ T cells from arresting along cerebral vasculature and rescues mice from fatal pathology and disease.

### Antigen presentation by cerebral ECs leads to PbA-specific CD8 T cell arrest and fatal disease

**Fig 8:**
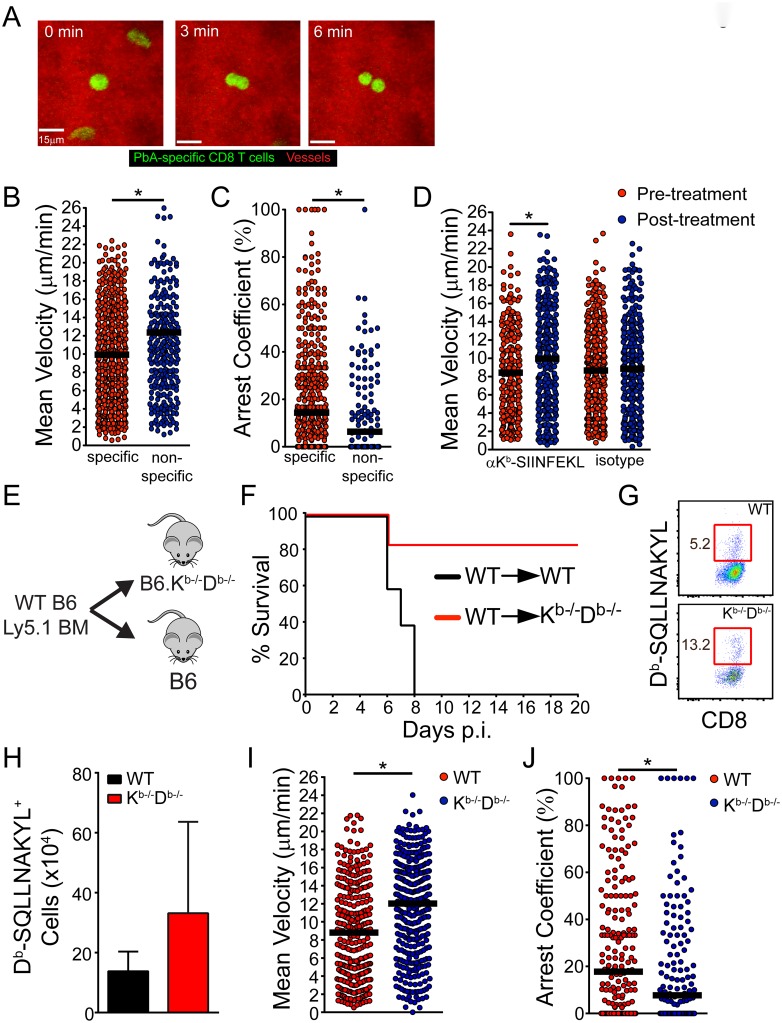
Fatal PbA-specific CD8^+^ T cell interactions with cerebrovascular ECs are cognate peptide MHC I dependent. (A) Representative maximal projections of a 3D time lapse shows a PbA-specific CD8^+^ T cell (green) dividing along the luminal surface of a cerebral blood vessel (red) at d6 p.i. The mouse was seeded with 10^4^ naïve mCerulean^+^ OT-I cells and infected with PbA-OVA-mCherry. See corresponding S10 Movie. (B and C) Quantification of PbA-specific CD8^+^ T cell velocities (B) and arrest coefficients (C) within the cerebral vasculature of d6 p.i. mice seeded with 10^4^ mCerulean^+^ OT-I cells and infected with PbA-OVA-mCherry. Intravenously injected YFP^+^ P14 cells served as the non-specific bystander control CD8^+^ T cells for this experiment. Each dot represents an individual T cell, and horizontal black bars denote the group mean. Data are representative of 7 independent experiments. (D) Quantification of PbA-specific CD8^+^ T cell velocities within the cerebral vasculature of d6 p.i. mice before and after i.v. injection of anti-K^b^-SIINFEKL or isotype control antibodies. Each dot represents an individual T cell, and horizontal black bars denote the group mean. Data are representative of 4 independent experiments. (E) Design of bone marrow (BM) chimeras used to collect the data shown in (F-J). (F) Survival curves of PbA-infected wild type (WT) into WT and WT into K^b-/-^D^b-/-^ BM chimeras (n = 5 per group; 2 independent experiments). (G) Representative flow cytometric plots depicting the frequency (red boxes and adjacent numbers) of D^b^-SQLLNAKYL-specific CD8 T cells in the spleens WT→WT and WT→K^b-/-^D^b-/-^ BM chimeras. Plots are gated CD45^+^ Thy1.2^+^ CD8^+^ cells. (H) Quantification of data from (G) (n = 3 per group; 2 independent experiments). (I and J) Quantification of PbA-specific CD8^+^ T cell velocities (I) and arrest coefficients (J) within the cerebral vasculature of WT→WT and WT→K^b-/-^D^b-/-^ BM chimeras at d6 p.i. Mice were seeded with 10^4^ naïve mCerulean^+^ OT-I cells and infected with PbA-OVA-mCherry. Each dot represents an individual T cell, and horizontal black bars denote the group mean. Data are representative of 7 independent experiments. Asterisks denote statistical significance (*P < 0.05).

During the development of ECM, we routinely observed PbA-specific CD8^+^ T cells dividing following arrest on the luminal surface of cerebral vasculature (Fig 8A; S10 Movie). Because cognate peptide-MHC I interactions can advance the cell cycle program of effector CD8^+^ T cells [55], we hypothesized that PbA-specific CD8^+^ T cells interact with the brain vasculature in an antigen dependent manner. To address this hypothesis, we first compared the dynamics of PbA-specific vs. bystander CD8^+^ T cells of an irrelevant specificity in cerebral blood vessels. Mice were seeded with mCerulean^+^ OT-I T cells and then infected with PbA-OVA. When these mice became symptomatic on day 6 post-infection, we intravenously injected yellow fluorescent protein (YFP)^+^ D^b^GP_33-41_ CD8^+^ T cells (YFP^+^ P14) purified from the spleens of a separate group of mice infected 8 days earlier with lymphocytic choriomeningitis virus (LCMV). YFP^+^ P14 cells were used as activated bystander CD8^+^ T cells because they are specific to the LCMV glycoprotein (GP), not PbA. TPM imaging and subsequent analysis of these two CD8^+^ T cell populations revealed that PbA-specific CD8^+^ T cells moved at a significantly slower speed (Fig 8B) and spent more time arrested along cerebral vasculature (Fig 8C and S6A Fig) than the bystander CD8^+^ T cells. These results demonstrate that antigen-specificity dictates the interaction between CD8^+^ T cells and brain microvasculature during ECM.

To further demonstrate the specificity of PbA-specific CD8^+^ T cell interactions, we used TPM to evaluate the dynamics of these cells following injection of an anti-peptide MHC I blocking antibody. PbA-OVA infected mice seeded mCerulean^+^ OT-I cells were imaged by TPM on day 6 post-infection. Midway through the imaging session, mice were injected i.v. with anti-K^b^-SIINFEKL (the peptide MHC complex recognized by OT-I cells) or isotype control antibodies. Injection of anti-K^b^-SIINFEKL, but not isotype, control antibodies significantly elevated the velocity PbA-specific CD8^+^ T cells (Fig 8D), further supporting that the interactions with cerebral ECs are antigen-specific.

Next, we set out to determine the functional importance of PbA-specific CD8^+^ T cell interactions with cerebral ECs during the development of ECM. This was accomplished by generating bone marrow (BM) chimeras in which MHC I deficient hosts (K^b-/-^D^b-/-^ mice) were reconstituted with wild type bone marrow (Fig 8E). These mice were incapable of presenting MHC I peptides on ECs and other stromal cells while maintaining normal hematopoietic presentation. Irradiated wild type mice receiving wild type bone marrow served as a control for this experiment. Interestingly, the K^b-/-^D^b-/-^ chimeras were nearly all resistant to fatal ECM, whereas the wild type controls succumbed to disease as expected (Fig 8F and S6C Fig). Protection from ECM was observed in K^b-/-^D^b-/-^ mice despite normal parasitemia levels (S6B Fig) and generation of an equal, if not greater, PbA-specific CD8^+^ T cell response relative to the wild type controls (Fig 8G and 8H). To evaluate how MHC I deficiency affected PbA-specific CD8 T cell interactions with the brain vasculature during ECM, we seeded wild type and K^b-/-^D^b-/-^ BM chimeras with mCerulean^+^ OT-I cells and then used TPM to monitor their intravascular motility 6 days following infection with PbA-Ova. Quantification of PbA-specific CD8^+^ T cells in the cerebral vasculature of the K^b-/-^D^b-/-^ BM chimeras revealed significantly increased velocities (Fig 8I) and reduced arrest within the lumen of brain blood vessels (Fig 8J and S6D Fig) relative to the wild type controls. Because there are no other radio-resistant cells within the vascular lumen, these data suggest that PbA-specific CD8^+^ T cells engage cerebral ECs in an antigen-dependent manner during ECM.

**Fig 9:**
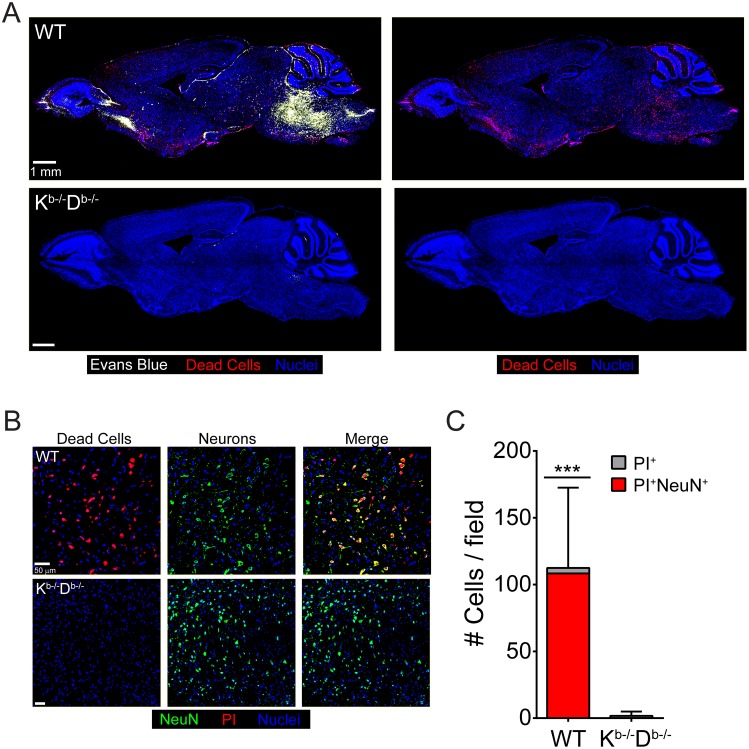
Brainstem pathology is absent in K^b-/-^D^b-/-^ BM chimeras. (A) Representative confocal images of sagittal brain sections from a wild type BM chimera mouse (top) or a K^b-/-^D^-/-^ BM chimera (bottom) at d6 p.i. following simultaneous i.v. injection of Evans blue (white) and propidium iodide (red) to visualize vascular leakage and cell death, respectively (n = 4 mice per group). Cell nuclei are shown in blue. (B) Representative confocal images of the brainstem from a wild type BM chimera mouse (top) or a K^b-/-^D^-/-^ BM chimera (bottom) at d6 p.i. following propidium iodide (red) injection and immunohistochemical staining for NeuN^+^ neurons (green). Cell nuclei are shown in blue. (C) The bar graph shows quantification of data shown in (B). Each bar represents the total number of brainstem cells that are PI^+^ (gray), and within the bar, the red coloration shows the proportion that are also NeuN^+^ (mean ± SD; n = 4 mice per group; 1 brain section per mouse; 4 images per brain section). Asterisks denote a statistically significant difference from all other groups (***P < 0.05).

Because PbA-infected K^b-/-^D^b-/-^ BM chimeras were resistant to ECM, we set out to determine whether they were also free from brainstem pathology normally associated with this disease (Fig 4A, 4D and 4E). Brains from wild type BM chimeras injected with PI and Evans blue at the peak of disease revealed extensive vascular leakage and cell death in the brainstem (Fig 9A). However, K^b-/-^D^b-/-^ BM chimeras were free of pathology at this same time point (Fig 9A). Nearly all of the dead cells in the brainstems of wild type BM chimeras were neurons, whereas brainstem neurons in K^b-/-^D^b-/-^ BM chimeras were unaffected (Fig 9B and 9C). These results suggest that antigen presentation by brain ECs, which fosters increased interactions with PbA-specific CD8 T cells, leads to severe brainstem pathology during ECM.

## Discussion

The pathological mechanisms underlying HCM are not entirely understood. Because ECM shares many of the pathological features of HCM, we set out to uncover novel mechanistic insights into the immunopathogenesis of this disorder. We made several important observations that significantly advance our understanding of cerebral malaria. Studies have shown that CM in humans and rodents is associated with BBB breakdown, edema, and hemorrhaging. During the peak of ECM, we noted that the meninges in addition to the brain parenchyma show evidence of profound vascular pathology, and this was associated a reduction in tight junction protein expression. Importantly, death from ECM is linked to marked vascular leakage and neuronal cell death in the brainstem, which is consistent with the edema and subsequent cerebral herniation recently observed in children with HCM [9]. Mechanistically, we uncovered that this fatal disease is caused by the activities of parasite-specific CD8^+^ T cells operating along cerebral blood vessels. As the disease developed, cerebrovascular ECs were highly activated by IFNγ, which promoted induction of cell adhesion and antigen presenting molecules. This in turn facilitated cognate peptide-MHC I dependent engagement by parasite-specific CD8^+^ T cells primarily on the luminal surfaces of cerebral blood vessels. The pathological significance of these interactions was demonstrated in mice rendered genetically deficient in their ability to present antigen in MHC I on ECs. These mice had reduced cerebrovascular engagement by CD8^+^ T cells and were resistant to fatal disease. Lastly, therapeutic administration of antibodies specific for VLA-4 and LFA-1 rapidly displaced CD8^+^ T cells from cerebral blood vessels and promoted survival, thus providing a simple yet effective means to treat this disease.

One of the most interesting findings in our study is the pathology observed in mice with severe ECM. By simultaneously injecting Evans blue and propidium iodide, we were able to evaluate the relationship between vascular leakage and cell death in mice succumbing to ECM. While vascular leakage was notable through the brain and meninges, the most striking areas of pathology were the olfactory bulb and brainstem. Vascular pathology was previously reported in the olfactory bulb of mice with ECM and linked to a decline in their sense of smell [56]. The severe pathology observed in the brainstem, however, is more relevant from the perspective of survival. Profound vascular leakage and neuronal cell death was seen in all mice succumbing to ECM and was associated with reduced expression of the tight junction protein, claudin-5. Sudden neuronal depolarization and death in this brain region would cause cardiorespiratory failure as observed in other neurological disorders [57, 58]. Importantly, a recent magnetic resonance imaging (MRI) study revealed evidence of severe brain swelling in 84% of children with HCM [9]. Brainstem herniation was also demonstrated in fatal instances of this disease. Our ECM results are consistent with brainstem herniation and pathology being the cause of death, which is supported by a MRI study showing significant displacement of the cerebellum and brainstem in mice with fatal ECM [48]. Therefore, increased intracranial pressure leading to cerebral herniation is the likely cause of death in rodents and children with CM.

To gain insights into the mechanisms that give rise to fatal edema during CM, we examined the activities of innate and adaptive immune cells. Our flow cytometric and two-photon imaging data revealed that ECM is associated with recruitment of innate and adaptive immune cells to blood vessels in the meninges and brain parenchyma. A strong consensus exists in the literature among several studies showing that CD8^+^ T cells play an essential role in ECM pathogenesis [21, 22, 31, 33, 34, 37, 38], and our data support this conclusion. There is, however, some debate regarding the role of innate immune cells, such as monocytes and neutrophils, in this disease. As mice developed ECM, our intravital time lapses revealed myelomonocytic cells migrating along cerebral blood vessels and acquiring adherent iRBCs. These cells were associated on occasion with vascular leakage; however, depletion had no impact on BBB breakdown, edema, or survival. This is different from the significant vascular disruption induced by synchronously extravasating myelomonocytic cells during fatal viral meningitis [42]. Some studies have implicated myelomonocytic cells in the pathogenesis of ECM [18, 27–29], whereas others have not [21, 33, 35, 36]. The difference in the outcome of these studies could be linked to many different variables, including strain of mice, depletion strategy, inadvertent blockade of T cells, and genetic variation in the strain of *Plasmodium* used. Regardless of the explanation, our findings suggest that while myelomonocytic cells may contribute to disease, they are not a dominant participant like CD8^+^ T cells.

At the peak of ECM, we observed parasite-specific CD8^+^ T cells slowly rolling, arresting, and dividing within the lumen of CNS vasculature. These interactions were highly associated with breakdown of the BBB and the flow of vascular contents into the meninges and parenchyma. Several lines of investigation have suggested that CTL-mediated killing of cerebrovascular ECs might be the cause of CNS vascular breakdown during ECM. Mice deficient in CTL effector pathways such as perforin and granzyme B are resistant to ECM [22, 37], apoptotic ECs have been identified in the retina [59] and brain [60, 61] during disease development, and CD8^+^ T cells isolated from ECM mice kill parasite-loaded ECs *in vitro* [62]. However, using a sensitive *in vivo* approach to quantify cell death, we were unable to demonstrate evidence of widespread EC death in the brains of highly symptomatic mice, but were able to show that Evans blue leakage was associated with reduced expression of tight junction proteins. This is consistent with other studies showing minimal EC death during ECM [17, 39]. Therefore, it is unlikely that CD8 T cells mediate ECM by directly killing brain ECs. Alterations in tight junction protein expression are the most likely explanation for vascular leakage in this model, although the low percentage of EC death we observed could certainly contribute to vascular leakage and surrounding brain pathology.

From the standpoint of vascular pathogenesis, we favor a mechanism whereby CD8^+^ T cells induce reversible alterations in EC tight junctions (such as claudin-5) that cause cerebrovascular leakage during ECM. A previous study demonstrated that CD8^+^ T cells can traverse the BBB following recognition of cognate peptide MHC I complexes on the lumen of cerebral ECs [63]. It was discovered more recently that CD8^+^ T cells can actually use granzyme B in a nontraditional manner to cleave vascular basement membrane [64]. This mechanism allows CD8^+^ T cells to extravasate across vasculature. Considering that we and others [39] have found parasite-specific CD8^+^ T cells along CNS vasculature during ECM, it is possible that the cumulative vascular breaks associated with CD8^+^ T cell extravasation contribute to global breakdown of the BBB. This event would be mitigated in granzyme B knockout mice, which are resistant to ECM [37].

CD8^+^ T cells could also use IFNγ to remodel the CNS vasculature. Studies using *in vitro* cultured ECs have shown that IFNγ causes cytoskeletal rearrangement and decreased barrier integrity [65, 66]. During HSV-2 infection, T cell-derived IFNγ has been shown to open the BBB, facilitating antibody access to the CNS [67]. Therefore, PbA-specific CD8^+^ T cells engaging CNS ECs in an antigen-specific manner could induce barrier openings through IFNγ release. In addition, IFNγ can promote cross presentation of antigen by cerebral ECs [62] as well as production of CXCL9 and 10 [68], chemokines known to attract CD8^+^ T cells. Thus, it is conceivable that engagement of peptide MHC I complexes by CD8^+^ T cells on cerebral ECs results in IFNγ release that opens tight junctions and recruits additional parasite-specific T cells. This type of amplification loop localizing primarily to cerebral vasculature would result in a rapid deterioration of the CNS barrier system. Consistent with this theory, deletion of IFNγ significantly reduced accumulation of parasite-specific CD8^+^ T cells along cerebral vasculature and completely eliminated fatal brainstem pathology.

The precise localization of parasite-specific CD8^+^ T cells to cerebrovascular ECs led us to consider whether this pattern was unique to the CNS. To address this question, we examined the vasculature of the ear skin as a representative peripheral location. Interestingly, our intravital imaging studies revealed that parasite-specific CD8^+^ T cells localized along brain but not ear vasculature. Moreover, vascular ECs extracted from the ear showed a significantly reduced expression of adhesion (ICAM-1 and VCAM-1) and antigen presenting (MHC I, MHC II) molecules when compared to cerebral ECs. This is an intriguing observation given that malaria is a systemic disease characterized by circulating iRBCs and inflammatory cytokines. Thus, vascular ECs throughout the body should have access to the same inflammatory mediators. The specific changes in cerebral ECs are best explained by parasite-specific T cell engagement following local antigen presentation. We observed upregulation of MHC I and II molecules on cerebral ECs, which would allow direct engagement by CD8^+^ and CD4^+^ T cells, respectively. These two T cell subsets are known to secrete IFNγ upon engagement and amplify the activities of one another during ECM [32]. Although we and others have shown that activation of the CNS endothelium occurs during ECM (Fig 6C) [69], we have provided direct evidence that this is driven by IFNγ(Fig 6D). Because antigen recognition occurs on cerebral ECs, it is likely that T cell-derived IFNγ plays a major role in the activation of the CNS vasculature. However, it remains to be determined why antigen presentation and T cell engagement occurs primarily on cerebral blood vessels and not vasculature residing in a peripheral site such as the ear. Features of the infecting parasite are also likely to contribute to severe disease. In children, one particular *var* gene product expressed on the iRBC surface to endothelial protein C receptor (EPCR) is associated with CM [70, 71], and brain autopsies of Malawian children who died from CM showed loss of EPCR at sites of sequestered iRBCs [72]. In mice, ECM-inducing *Pb*A differs by only 18 non-synonymous mutations in open reading frames from the NK65 parasite that does not cause ECM, suggesting that these parasite genes may contribute to the unique brain pathology during ECM.

The localization of parasite-specific CD8^+^ T cells along cerebral vessels [39] does not alone prove that these cells are engaged in cognate peptide MHC I interactions or that these interactions are important for disease development. To demonstrate the specificity of these interactions, we conducted several lines of investigation. We demonstrated previously that CTL division could be advanced by peptide MHC I interactions in the virally infected meninges [55]. Interestingly, we observed a similar pattern of parasite-specific CD8^+^ T cell division in the lumen of cerebral blood vessels during ECM, suggesting antigen-specific interactions. The specificity of the vascular CD8^+^ T cell interactions was proven by injecting anti-K^b^-SIINFEKL antibodies intravenously during ECM. This increased the velocity of luminal parasite-specific CD8^+^ T cells, indicating that the interactions with ECs were in fact antigen specific. This conclusion was further supported by the preferential arrest of parasite-specific CD8^+^ T cells on cerebral vasculature when compared to bystander CD8^+^ T cells of an irrelevant specificity. Lastly, the functional importance of the interactions was demonstrated by removing MHC I from the stromal compartment through the generation of bone marrow chimeras. Expression of MHC I on the hematopoietic system but not stromal cells such as vascular endothelium significantly reduced parasite-specific CD8^+^ T cell arrest on cerebral ECs and promoted survival. *In vitro* studies have shown that human and murine brain ECs have the ability to internalize and cross-present *Plasmodium* antigen to CD8^+^ T cells [41, 62]. In fact, *ex vivo* cultured brain ECs from mice with ECM have the capacity to stimulate CD8^+^ T cells in an antigen-specific manner. These data collectively demonstrate that CD8^+^ T cells mediate fatal vascular pathology during ECM via antigen-dependent interactions with cerebrovascular ECs.

While murine and human CM share similar pathologies, the role of CD8^+^ T cells in HCM remains unknown. Less attention is given to CD8^+^ T cells during HCM because they are difficult to find in human post-mortem brain samples [73]. It should be noted, however, that we and others [21] have also found it difficult to identify intravascular CD8^+^ T cells in post-mortem brain samples from mice with ECM. An inability to observe an abundance of these cells histologically does not negate their involvement in HCM. It is unfortunately not possible to examine parasite-specific CD8^+^ T cells intravitally during HCM (as we have done in mice); however, genetic studies offer some clues regarding their involvement in this disease. For example, resistant vs. susceptibility to HCM has been linked to specific human leukocyte antigen class I alleles [74, 75]. In addition, the CD8 T cell chemoattractant, CXCL10, is a strong biomarker for HCM [76–78], and a genetic polymorphism that elevates CXCL10 levels was linked to an increased incidence of HCM [79]. Importantly, CXCL10 blockade or deficiency is partially protective against development of ECM [25, 26]. It remains to be determined whether CD8^+^ T cells are definitively involved in the pathogenesis of HCM, but further studies are warranted given the similarities between HCM and ECM.

Without knowing the specificity of disease-inducing parasite-specific T cells, it would be difficult to therapeutically target these cells during CM. Thus, we surmised that a non-specific displacement of T cells from the cerebral vasculature would provide a more effective means to thwart this disease. We demonstrated that late blockade with antibodies against LFA-1 and VLA-4 completely prevented development of ECM, which is consistent with other studies showing the effectiveness of adhesion molecule blockade in this model [17, 53, 54]. However, the mechanism by which this therapy prevented disease was unclear. Using intravital imaging, we observed that anti-adhesion molecule therapy completely disengages CD8^+^ T cells from the CNS vasculature at the peak of disease without affecting the number of extravascular CD8^+^ T cells. These data indicate that this disease can be treated by interfering with luminal interactions between CD8^+^ T cells and cerebrovascular ECs. Natalizumab (anti-VLA-4) and Efaluzimab (anti-LFA-1) are both FDA approved drugs used to treat human inflammatory diseases [80–82], opening the possibility of an expedited intervention in patients with HCM. It would be important to begin treatment in patients with edema prior to the development of cerebral herniation, which would result in irreversible brainstem pathology.

In conclusion, we have provided the first *in vivo* evidence detailing how CD8^+^ T cells cause ECM. Parasite-specific CD8^+^ T cells engage CNS ECs in an antigen-dependent manner, which leads to profound vascular breakdown (associated with alterations in tight junction protein expression), edema, loss of brainstem neurons, and subsequent death. Despite CTL engagement, we observed little evidence of EC death during ECM. We therefore hypothesize that CD8^+^ T cells use a noncytopathic mechanism to disrupt cerebrovascular EC tight junctions. This signifies that the disease is reversible up to the point when severe edema gives rise to cerebral herniation and death of brainstem neurons (an irreversible event). The reversibility of the disease is demonstrated by the effectiveness of late anti-adhesion molecule blockade. Because many patients with CM likely arrive in the hospital with active BBB breakdown, effective therapies need to target parasite acquisition by ECs and subsequent CD8^+^ T cell engagement. In addition to anti-malarial drugs and supportive care, consideration should also be given to therapeutics that interfere with T cell function / metabolism [83, 84] or that temporarily displace these cells from cerebral vasculature (e.g. anti-VLA-4 / LFA-1). A reduction of parasite burden in combination with a transient disruption of T cell activity should give the BBB sufficient time to repair and for neurological disease to subside.

## Materials and Methods

### Mice

C57BL/6J (B6), B6.129S7-*Ifn* ^*tm1Ts*^/J (IFNγ^-/-^), B6.129S4-Ccr2^tm1Ifc^/J (CCR2^-/-^) B6.129P-Cx3cr1tm1Litt/J (CX3CR1^gfp/gfp^)[85], B6.SJL-Ptprca Pepcb/BoyJ (Ly5.1), and BL/6-Tg(TcraTcrb)1100Mjb/J (OT-I) mice were purchased from The Jackson Laboratory (Bar Harbor, ME). CX3CR1^gfp/gfp^, OT-I, and Ly5.1 were then bred and maintained under specific pathogen free conditions at the National Institute of Health (NIH). B6 D^b^GP_33–41_ TCR-tg (P14) [86], B6 LysM^gfp/+^ [44], B6 H-2K^b-/-^D^b-/-^, B6 actin-mCerulean, and B6 actin-YFP were also bred and maintained at the NIH. CX3CR1^gfp/+^ mice were generated by crossing B6 mice with CX3CR1^gfp/gfp^ mice. Actin-mCerulean and actin-YFP mice were made as described below. YFP^+^ P14 and mCerulean^+^ OT-I mice were derived from the following F1 crosses: actin-YFP x P14 and actin-mCerulean x OT-I, respectively. All mice bred in house were confirmed to be on a pure C57BL/6J background by SNP analysis (Charles River).

### Transgenic mouse generation

All transgenic mice were generated by the National Institute of Mental Health (NIMH) Transgenic Core Facility. Transgenic mice expressing monomeric cerulean (mCerulean) under the chicken β-actin promoter were generated as described [87] by first PCR amplifying the entire 717 bp mCerulean coding region from the pCMV-mCerulean vector (Addgene) using primers (fwd: 5’ATATATGAATTCGCCACCATGGTGAGCAAGGGCGAGG3’; rev: 5'ATATATCTC GAGTTACTTGTACAGCTCGTCCATG3') containing EcoRI and XhoI restriction sites. This cDNA was cloned into the same sites following removal of the GFP sequence from the pCAG-GFP vector (Addgene). The resultant plasmid was digested with Sal I / HindIII, and a 2925 bp fragment containing the CMV early enhancer/chicken β-actin (CAG) promoter, mCerulean, and PolyA sequence was prepared for microinjection into the pronuclei of fertilized mouse eggs. Mice expressing the Venus variant of yellow fluorescent protein (YFP) under the CAG promoter were generated in a similar manner. The entire 717 bp coding sequence for YFP was PCR amplified using the aforementioned primers and cloned into pCAG-GFP following removal of GFP with EcoRI and XhoI. A 2925 bp fragment consisting of the CAG promoter, YFP, and a PolyA sequence cut with Sal I / HindIII and prepared for microinjection. To generate all transgenic mice, linearized constructs were injected into C57BL/6J eggs. Following selection of transgene positive founder lines, all mice were backcrossed one additional generation onto C57BL/6J background before intercrossing.

### Parasites and infections

PbA was maintained and used as previously reported [83]. PbA.OVA::mCherry_hsp70_ (PbA-OVA-mCherry) parasites were kindly provided by C. Janse and S. Khan [45]. PbTg (PbA-OVA-GFP) parasites were kindly provided by W. Heath [38]. All mice were infected intraperitoneally (i.p.) with 10^6^ parasitized red blood cells (pRBCs). Parasitemia was determined by Giemsa-stained thin blood smear or by flow cytometry as described [88]. Mice were monitored daily for neurological symptoms of ECM using a quantitative scale as described [40]. For survival studies, mice were euthanized upon reaching a score of 0. For LCMV infections, mice were injected with 10^5^ plaque forming units of LCMV Armstrong 53b strain i.p.

### BBB integrity assay

Mice were i.v. injected with 20mg Evans blue (Sigma) per kg body weight at the indicated time points p.i. After 4 hours, brains were extracted following saline perfusion and then processed as described previously [42].

### Brain water content assay

Brain water content was measured as previously described [19]. Weights of brains removed at indicated time points were compared to the dry weight after overnight incubation at 80°C.

### Immunohistochemistry

Mice were anesthetized with chloral hydrate and perfused with 4% paraformaldehyde in PBS for H&E analysis, 10% neutral buffered formalin (NBF) for meningeal whole mounts, or 2% NBF in PBS for all other immunohistochemical stains. For meningeal whole mounts, skull caps were removed and incubated overnight in 10% NFB. Following a brief wash, meninges (including dura, arachnoid, and partial pia layers) were carefully removed from the bone with fine-tipped forceps and placed in PBS + 2% fetal bovine serum (FACS buffer) at 4°C and stained with primary antibody overnight. After a brief wash, the meninges were stained with secondary antibodies for 1 hr at room temperature, followed by another brief wash and then a DAPI stain for 5 min. The meninges were placed in mounting medium on a glass slide, spread out and flattened with forceps, and cover-slipped. Brain tissues were extracted following perfusion and incubated either 2 hours for TJ stains or overnight for all other stains in the same solution with which they were perfused. After a brief wash in PBS, brain tissues were incubated in 30% sucrose in PBS for 24 hours. H&E staining was performed by HistoServe, Inc. For all other stains, tissues were frozen in Tissue-Tek optimal cutting media (Thermo Fisher Scientific). A Leica CM1850 cryostat was used to cut 40 μm thick tissue sections for TJ staining or 20 μm sections for all other stains. Primary stains were performed overnight at 4°C, and secondary and tertiary stains were performed for 1 hr at room temperature with 3 x 5 min washes after each stain. DAPI (Sigma) was added for 5 min at RT to label cell nuclei. Following staining, 1 drop of FluorSave Reagent (Calbiochem) was added to each section before addition of a coverslip. The following primary antibodies were used: anti-Claudin-5 Alexa Fluor 488 (4C3C2) (Thermo Fisher Scientific) (1:100), polyclonal anti-laminin (Abcam) (1:200), anti-NeuN (A60) (EMD Millipore) (1:250), polyclonal anti-PECAM-1 (CD31) (EMD Millipore) (1:40), and anti-TER119 (Biolegend) (1:200). All secondary antibodies and staining reagents used for immunohistochemistry were purchased from Jackson ImmunoResearch including anti-rat Alexa Fluor 647, anti-goat Rhodamine Red-X, biotin anti-goat, biotin anti-rabbit, streptavidin Alexa Fluor 488, and streptavidin Rhodamine Red-X. All secondary antibodies were used at a concentration of 1:500 with the exception of streptavidin conjugates, which were used at a concentration of 1:1000. H&E images were acquired using a Nikon Eclipse Ci microscope with 4x/0.2 NA and 40x/0.75 objectives. Fluorescent images were acquired using an Olympus FV1200 laser scanning confocal microscope equipped with 405, 458, 488, 515, 559, and 635 laser lines, 4 side window PMTs for simultaneous 4 channel acquisition, and 4x/0.16 NA, 10x/0.4 NA, 20x/0.75, 40x/0.95, and chromatic aberration corrected 60x/1.4 NA objectives. For cell death analyses, 10–12 0.4 mm^2^ field images were collected from the brain sections of each mouse. Each field was chosen at random within one of four brain regions: olfactory bulb, cerebrum, cerebellum, and brainstem. Images were analyzed using Imaris 7.6.4 software. Only cells with PI staining throughout the nucleus were counted as dead. To determine co-localization of PI and NeuN, PI^+^ nuclei were first identified and labeled using the spots function with Imaris software. Each spot was then masked in the NeuN channel to reveal all the NeuN^+^PI^+^ co-staining. For tight junction analysis, areas of vascular hemorrhaging were first identified within brain sections of symptomatic mice at d6 p.i. by Evans blue staining. We focused specifically on the frontal cortex, brainstem, and cerebellum. Within those regions, *xyz* images (each 808,992 μm^3^) were captured by confocal microscopy from each brain region per mouse. Three to four *z*-stacks were captured from areas with and without Evans blue leakage. Images from the corresponding brain regions of uninfected mice were also collected. Within each group, we then quantified the intensity of claudin-5 expression on 30–32 blood vessels as described previously [89]. Briefly, using Imaris 7.6.4 software, contours were generated around individual blood vessels based on CD31 staining and used to create a 3D surface. The claudin-5 intensity per unit area of an individual blood vessel within this surface was then calculated as follows: (total # voxels x claudin-5 MFI) / total surface area of vessel.

### *In vivo* cell death assay

Mice were injected with 200μg Evans blue dye i.v. for one hour and 50μg propidium iodide (Invitrogen) i.v. for 30 minutes. Mice were anesthetized, perfused, and brain tissue was processed as described above.

### Mononuclear cell isolation

Anesthetized mice received an intracardiac perfusion with PBS to remove all blood leukocytes other non-adhered cells. Single-cell spleen suspensions were prepared by mechanical disruption through a 100 mm mesh barrier followed by RBC lysis with Ack lysis buffer (0.15M NH_4_Cl, 10mM KHCO_3_, 0.1mM EDTA). Leukocytes were isolated from the meninges, using forceps to gently separate them from the underside of skull cap (exactly as performed for immunohistochemistry whole mounts) followed by enzymatic digestion in 2mg/ml collagenase D (Roche) + 50 mg/ml DNase (Roche) in RPMI for 30min at 37°C. Leukocytes were isolated from the brain as described [90]. EC isolation was adapted from [91]. Briefly, whole meninges and minced brains from perfused mice were enzymatically digested in 0.8mg/ml Dispase II + 0.2mg/ml Collagenase P + 0.1mg/ml DNase (all Roche) in RPMI at 37°C for 15 min with gentle shaking followed by 15 min of mechanical comminution at 37°C. Following digestion, supernatants were isolated and washed. Meningeal ECs were ready for staining but brain EC preps were resuspended in a 40% Percoll (GE Healthcare) gradient in HBSS and centrifuged to remove excess myelin.

### Flow cytometry

Surface staining and Fc blocking was performed as described [90]. Dead cells were excluded from the analysis by using the LIVE/DEAD fixable Blue Cell Staining kit (Invitrogen). The following antibodies and reagents from BioLegend were used: CD4 PE (RM4-5), CD8a APC (53–6.7), CD8β.2 FITC (53–5.8), CD11b Brilliant Violet 605 (M1/70), CD31 PE/Cy7 (390), CD45.1 AlexaFluor 647 (A20), CD45.2 Alexa Fluor 700 (104), CD45.2 FITC (104), CD54 Alexa Fluor 488 (YN1/1.7.4), CD106 biotin (429), Gr-1 PE (RB6-8CJ), H-2D^b^ Alexa Fluor 647 (KH95), H-2K^b^ PerCP/Cy5.5 (AF6-88.5), IA^b^/IE^b^ Pacific Blue (M5/114.15.2), Ly6C APC/Cy7 (HK1.4), Ly6G PE (1A8), Streptavidin Brilliant Violet 605, Thy1.2 Alexa Fluor 700 (30-H12), and corresponding isotype controls. To identify PbA-specific CD8^+^ T cells, single cell suspensions were first stained with H-2D^b^-SQLLNAKYL^+^ pentamers (ProImmune) in PBS + 10% BSA for 10 min at room temperature before surface staining as described above. PbA-specific CD8^+^ T cells were also identified by IFNγ production following *in vitro* stimulation with SQLLNAKYL peptide. Briefly, 2 x 10^6^ splenocytes from the denoted mice were incubated with 1μg/ml SQLLNAKYL peptide (AnaSpec), 100U/ml IL-2 (NIH), and 10μg/ml BFA (Sigma) in RPMI complete media at 37°C for five hours. To detect intracellular IFNγ production, single cell suspensions were surface stained as described above, treated with cytofix/cytoperm (BD), and then stained intracellularly with anti-IFNγPE/Cy7 (XMG1.2) (BioLegend). Samples were acquired using an LSRII flow cytometer (BD), and data were analyzed using FlowJo software version 9.7.2 (Tree Star).

### Cell depletion/blocking studies

All antibodies used for cell depletion and blocking assays were purchased from BioXcell. Mice were depleted of neutrophils by injecting 500 μg of anti-Ly6G (clone 1A8) i.p. on days -1 and 3 p.i. Mice were depleted of CD4 or CD8^+^ T cells by i.p. injection of 500 μg (clone GK1.5) or 200 μg anti-CD8 (clone YTS 169.4), respectively, on d4 p.i. Cell depletion efficiency was calculated in the blood using the following formula: 100 –((frequency of targeted cell population of a mouse / the average frequency of the targeted cell population of the 4–5 untreated mice) x 100). For adhesion molecule blocking assays mice were treated with a combination of 500 μg anti-LFA-1 (clone M17/4) and 500 μg anti-VLA-4 (clone PS/2) i.v. at the indicated time points. For cognate peptide-MHC blocking assays, mice were treated with 680 μg anti-Kb-SIINFEKL (clone 25-D1.16) or mIgG1 isotype control (clone MOPC-21) antibodies i.v. at the indicated time points.

### Adoptive transfers

Mice were seeded i.v. with 10^4^ mCerulean^+^ OT-I or YFP^+^ P14 CD8^+^ T cells purified from the splenocytes of naïve transgenic mice using a CD8 negative selection kit (Stem Cell Technologies). To compare activated PbA-specific and non-specific CD8^+^ T cell responses in the same mice, activated YFP^+^ P14s were first purified from the splenocytes of previously seeded mice using a CD8 positive selection kit (Stem Cell Technologies), 8 days following infection with LCMV. Symptomatic PbA-infected mice previously seeded with mCer OT-1 cells were then i.v. injected with 5x10^6^ activated YFP-P14s i.v.

### Two-photon microscopy

Mice were anesthetized and thin skull windows were prepared as previously described [42, 43]. Mice were injected with 5 μl Qdot655 (BD) or 50 μg Evans blue where indicated to visualize blood vessels. 4D datasets were acquired using an SP5 two-photon microscope (Leica) equipped with two Mai Tai HP DeepSee lasers (SpectraPhysics), an 8,000-Hz resonant scanner, a 20×/1.0 NA objective, an NDD4 detector array, and a custom-environment chamber. Simultaneous excitation and detection of multiple fluorophores was achieved using custom dichroic mirrors (Semrock) and by tuning one laser to 905 nm and the other to 990 nm. All imaging studies focused on the meninges and superficial neocortex. Imaging data were processed with Imaris 7.6.4 software. A surface was created for each CNS blood vessel and PbA-specific CD8^+^ T cells located within that surface (vessel lumen) were quantified, whereas T cells residing outside the surface (perivascular or parenchymal) were quantified separately. Mean track velocities (μm/min), arrest coefficients (proportion of time a cell spent arrested <2 μm/min), and arrest duration (the total amount of time a cell spent arrested <2 μm/min) were calculated for all individual luminal PbA-specific CD8^+^ T cell tracks using Imaris 7.6.4 and T Cell Analyzer software (TCA 1.7.0; Strathclyde Institute of Pharmacy and Biomedical Sciences). All time lapses used for these analyses were at least 40 minutes in length.

### Bone Marrow (BM) chimeras

BM was harvested from femurs and tibias of Ly5.1 mice and 5x10^6^ BM cells were i.v. injected into recipient mice following a lethal irradiation dose of 900 RAD. Mice received antibiotics in drinking water for 4 weeks following irradiation and were allowed 8 weeks to fully reconstitute bone marrow and donor peripheral cells.

### Statistical analysis

Statistical analyses for data were performed using a Student’s *t* test (two groups) or ANOVA (more than two groups) in Prism 6 (GraphPad Software). Groups were considered statistically different at a *p* value of <0.05. All data are displayed as the mean ± SD.

### Ethics statement

This study was carried out in strict accordance with the recommendations in the Guide for the Care and Use of Laboratory Animals of the National Institutes of Health. The protocol was approved by the NINDS Animal Care and Use Committee (Protocol Number: 1295–14).

## Supporting Information

S1 Fig Parasitemia, clinical score, and brain pathology in PbA-infected mice. (A) Blood parasitemia percentages and clinical scores were evaluated over time in wild type B6 mice infected with PbA (mean ± SD; n = 5 mice per group). (B) Representative brains from a naïve (left) and a PbA-infected mouse at d6 p.i. (right). Note the significant hemorrhaging observed in symptomatic mice at day 6. (C) Representative brains from a naïve (left) and a mouse at d6 p.i. (right) following i.v. injection of Evans Blue dye reveal evidence of profound vascular breakdown (blue coloration) during ECM. (D) Fluorometric quantification of data shown in (C). Data are represented as mean ± SD (n = 4–5 mice per group). (E) Quantification of brain water content from naïve and d6 p.i. mice (mean ± SD; n = 4 mice per group). (F) H&E stained sections from the brains of a naïve (top) and d6 p.i. (bottom) demonstrate evidence of perivascular hemorrhaging (black arrows) in the brain parenchyma during ECM. Boxed regions in left panels are magnified and displayed in right panels (n = 4 mice per group). BG = basal ganglia, CC = corpus callosum, AC = anterior commissure. (G) Confocal images from a representative sagittal brain section of a mouse infected 6 days earlier with PbA-OVA-GFP (n = 4 mice per group). The region within the white box is magnified and displayed in the bottom panel. Note the leakage of red blood cells (red) and PbA parasites (green) from blood vessels (white) into the parenchyma. Nuclei are shown in blue. All data in this figure are representative of two independent experiments. Asterisks denote statistical significance (*P < 0.05).
(TIF)

S2 Fig Myelomonocytic cell depletion and disease course in PbA-infected CCR2^-/-^ mice. (A) Representative flow cytometric dot plots showing wild type (top) and neutrophil-depleted CCR2-deficient mice (bottom). Plots are gated on Thy1.2^-^CD11b^+^ cells. The right panel depicts a graphical representation of the depletion efficiency in the blood based on the following calculation: 100 –((%Thy1.2^-^Cd11b^+^Gr-1^+^Ly6C^+^ cells in the blood of each treated mouse divided by the average %Thy1.2^-^Cd11b^+^Gr-1^+^Ly6C^+^ cells in the blood of wild type mice) x 100). Blood parasitemia percentages (B) and clinical score (C) in wild type vs. neutrophil-depleted CCR2^-/-^ mice at d6 p.i. (n = 5 mice per group; two independent experiments).
(TIF)

S3 Fig Depletion efficiencies, parasitemia levels, and brain pathology in mice with T cell deficiencies. (A) Representative flow cytometric dot plots showing depletion of CD8^+^ (top) and CD4^+^ (bottom) T cells in the blood. The right panel is a graphical representation of the depletion efficiency. (B) Blood parasitemia was quantified in the following d6 p.i. mice described in Fig 3A: untreated B6, B6 + anti-CD4, B6 + anti-CD8. (n = 4–5 mice per group). (C) Representative brains from wild type (left) and CD8^+^ T cell depleted (right) mice at d6 p.i. (n = 5 mice per group). Note the absence of vascular hemorrhaging in CD8^+^ T cell depleted mice. (D) Representative brains from wild type (left) and CD8^+^ T cell depleted (right) mice at d6 p.i. following i.v. injection of Evans Blue dye. (E) Fluorometric quantification of data shown in (D) (mean ± SD; n = 5 mice per group). Note the absence of BBB breakdown in CD8^+^ T cell depleted mice. (F) Quantification of brain water content from wild type and CD8^+^ T cell depleted mice at d6 p.i. (mean ± SD; n = 5 mice per group). All data in this figure are representative of two independent experiments.
(TIF)

S4 Fig Meningeal EC phenotype and disease course in IFNγ^-/-^ mice. (A) Representative flow cytometric dot plots show the gating strategy used to identify ECs in the CNS. (B) The bar graph depicts the geometric mean fluorescent intensity of the denoted molecules on meningeal ECs extracted from naïve, d4 p.i., d5 p.i., and d6 p.i. B6 mice (mean ± SD; n = 3–4 mice per group). (C) Survival curve showing PbA-infected wild type and IFNγ^-/-^ mice over time (n = 5 mice per group). (D and E) Blood parasitemia (D) and clinical scores (E) for the d6 p.i. wild type and IFNγ^-/-^ mice shown in Fig 6D (n = 5 mice per group). (F) Graphical representation of D^b^-SQLLNAKYL^+^ CD8 T cells in the spleen, blood, and brain of WT and IFNγ mice at d6 p.i. All data in this figure are representative of two independent experiments. Asterisks denote statistical significance (*P < 0.05).
(TIF)

S5 Fig ECM disease course following antibody-mediated adhesion molecule blockade. (A) Survival curve showing PbA-infected mice treated with 500 μg anti-VLA-4 + 500 μg anti-LFA-1 (red) or isotype control (black) antibodies i.v on day 5.5 and then again on day 6.5 p.i. Data are representative of three independent experiments with 5 mice per group. (B) Blood parasitemia levels in PbA-infected mice treated with 500 μg anti-VLA-4 + 500 μg anti-LFA-1 (red) or isotype control (black) antibodies i.v at day 5.5 and 6.5 p.i. (n = 5 mice per group; 3 independent experiments). (C) Clinical scores in the same mice assessed at d5.5, d6, and d12 p.i. (D) Representative dot plots of splenic CD8^+^ T cell IFNγ expression following *in vitro* stimulation with SQLLNAKYL peptide at d6 p.i. from mice treated with blocking or isotype control antibodies at d5.5 p.i. Plots are gated on live CD45^+^Thy1.2^+^CD8^+^ cells. (E) Quantification of data shown in (D) (mean ± SD; n = 5 mice per group). Data are representative of two independent experiments.
(TIF)

S6 Fig Disease course in WT and K^b-/-^D^b-/-^ BM chimeras. (A) Quantification of maximum arrest time for PbA-specific CD8 T cells within the cerebral vasculature of d6 p.i. mice before and after i.v. injection of anti-K^b^-SIINFEKL or isotype control antibodies. Each dot represents an individual T cell, and horizontal black bars denote the group mean. Data are representative of 4 independent experiments. (B) Blood parasitemia and (C) clinical scores in PbA-infected WT→WT and WT→K^b-/-^D^b-/-^ BM chimeras at d6 p.i. (n = 5 per group; 2 independent experiments). See corresponding Fig 8F. (D) Quantification of maximum arrest time for PbA-specific CD8^+^ T cells within the cerebral vasculature of WT→WT and WT→K^b-/-^D^b-/-^ BM chimeras at d6 p.i. Each dot represents an individual T cell, and horizontal black bars denote the group mean. Data are representative of 7 independent experiments. Asterisks denote statistical significance (*P < 0.05).
(TIF)

S1 Movie Dynamics of innate immune cells during ECM. Representative time lapses of 3D reconstructions show the distribution of LysM^gfp/+^ myelomonocytic cells (green) in the Evans blue-filled vasculature (red) of mice at d0, d5 p.i., and d6 p.i. Time lapses were captured in brain and ear. Separate mice were used at each time point. Note the steady accumulation of myelomonocytic cells within the vascular lumen of the brain and ear during the course of PbA infection.
(MOV)

S2 Movie Innate immune cell-associated vascular pathology in the brain during ECM. Representative time lapse of a 3D reconstruction showing LysM^gfp/+^ myelomonocytic cells (green) within the brain vasculature (red) of a PbA-infected mouse at d6 p.i. Vasculature was labeled with Evans blue. Note the dense accumulation of myelomonocytic cells and associated vascular leakage (denoted by white arrows) in a capillary and a post-capillary venule.
(MOV)

S3 Movie Intravascular phagocytosis of PbA by monocytes during ECM. Representative time lapse of a 3D reconstruction showing monocytes (green) and PbA parasites (red) in the Evans blue-filled vasculature (blue) of a neutrophil-depleted LysM^gfp/+^ mouse at d6 p.i. White circles highlight three areas where intravascular parasites (presumably inside RBCs) are enveloped by luminal monocytes.
(MOV)

S4 Movie Perivascular phagocytosis of PbA during ECM. Representative time lapse of a 3D reconstruction showing perivascular macrophages (yellow), PbA parasites (red), CD8^+^ T cells (green) in the brain of Evans blue (blue) injected mice at d6 p.i. Two different perivascular macrophages can be seen simultaneously projecting processes toward and enveloping a PbA parasite cluster.
(MOV)

S5 Movie Dynamics of PbA-specific CD8^+^ T cells. Representative time lapses of 3D reconstructions showing PbA-specific CD8^+^ T cells (green) in relation to the Evans blue-filled vasculature (red) of the brain and ear for a mouse at d6 p.i. Notice the absence of PbA-specific CD8^+^ T cells in the ear vs. brain vasculature. In addition, the vast majority of PbA-specific CD8^+^ T cells localize to the lumen of cerebral vasculature.
(MOV)

S6 Movie Vascular pathology associated with PbA-specific CD8^+^ T cell activity along cerebral vasculature. Representative time lapse of a 3D reconstruction showing PbA-specific CD8^+^ T cells (green) associated with the cerebral vasculature (red) of a mouse at d6 p.i. Part 1 shows significant leakage of Evans blue along two large blood vessels. This type of pathology is commonly observed in meningeal and brain vasculature during the peak of ECM. Part 2 of this video is the same as Part 1 except that the PbA-specific CD8^+^ T cells (green) are now visible. Note that the CTL are highly interactive with the lumen of cerebral vasculature, especially in areas of associated vascular leak.
(MOV)

S7 Movie Magnified view of PbA-specific CD8^+^ T cell-associated vascular pathology. Magnified view of a PbA-specific CD8^+^ T cell (green) migrating along a cerebral blood vessel (red) at d6 p.i. Note how the path (dotted line) of the highlighted PbA-specific CD8^+^ T cell (circle) is temporarily associated with vascular leakage.
(MOV)

S8 Movie Application of a volumetric mask to CNS blood vessels reveals anatomical location of PbA-specific CD8 T cells. A representative maximal projection of a 3D reconstruction shows the spatial relationship between mCerulean^+^ OT-I cells (green) and CNS blood vessels (gray) at d6 p.i. A volumetric mask was applied to the vascular signal corresponding intravenously injected Evans blue dye. Following application of the mask, only extravascular OT-I cells are visible.
(MOV)

S9 Movie PbA-specific CD8^+^ T cell dynamics following intravascular adhesion molecule blockade. Representative time lapse of a 3D reconstruction showing PbA-specific CD8^+^ T cells (green) in relation to the Evans blue-filled cerebral vasculature (red) of a d6 p.i. mouse before and after administration of anti-LFA-1 and anti-VLA-4 antibodies i.v. Note how antibody administration causes PbA-specific CD8^+^ T cells to become rapidly displaced from cerebral vasculature while perivascular and parenchymal cells remain unaffected.
(MOV)

S10 Movie PbA-specific CD8 T cells divide within cerebral vasculature during ECM. Representative time lapse of a 3D reconstruction showing a PbA-specific CD8^+^ T cell (green) in an Evans blue-filled cerebral blood vessel (red) of a mouse at d6 p.i. Note how the PbA-specific CD8 T cell in the center of the blood vessel (circle) divides and afterward the daughter cells migrate away and begin scanning the vascular lumen.
(MOV)
