# Supplementary figures and images for: CD8+ T Cells Induce Fatal Brainstem Pathology during Cerebral Malaria via Luminal Antigen-Specific Engagement of Brain Vasculature
Source: PLoS Pathog. 2016 Dec 1;12(12):e1006022. doi: 10.1371/journal.ppat.1006022 (PMC5131904; doi:10.1371/journal.ppat.1006022)

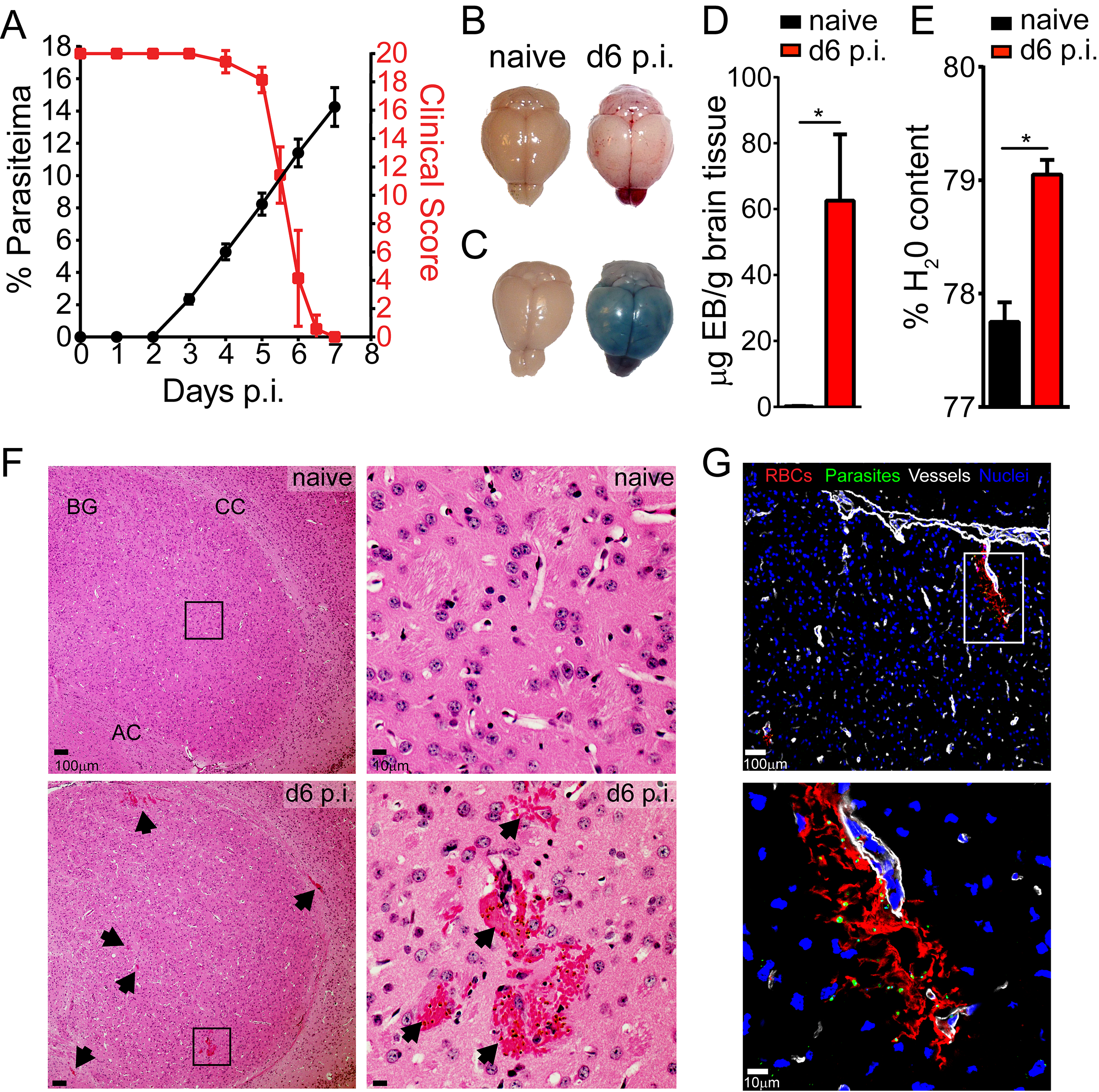

Supplement: S1 Fig — (A) Blood parasitemia percentages and clinical scores were evaluated over time in wild type B6 mice infected with PbA (mean ± SD; n = 5 mice per group). (B) Representative brains from a naïve (left) and a PbA-infected mouse at d6 p.i. (right). Note the significant hemorrhaging observed in symptomatic mice at day 6. (C) Representative brains from a naïve (left) and a mouse at d6 p.i. (right) following i.v. injection of Evans Blue dye reveal evidence of profound vascular breakdown (blue coloration) during ECM. (D) Fluorometric quantification of data shown in (C). Data are represented as mean ± SD (n = 4–5 mice per group). (E) Quantification of brain water content from naïve and d6 p.i. mice (mean ± SD; n = 4 mice per group). (F) H&E stained sections from the brains of a naïve (top) and d6 p.i. (bottom) demonstrate evidence of perivascular hemorrhaging (black arrows) in the brain parenchyma during ECM. Boxed regions in left panels are magnified and displayed in right panels (n = 4 mice per group). BG = basal ganglia, CC = corpus callosum, AC = anterior commissure. (G) Confocal images from a representative sagittal brain section of a mouse infected 6 days earlier with PbA-OVA-GFP (n = 4 mice per group). The region within the white box is magnified and displayed in the bottom panel. Note the leakage of red blood cells (red) and PbA parasites (green) from blood vessels (white) into the parenchyma. Nuclei are shown in blue. All data in this figure are representative of two independent experiments. Asterisks denote statistical significance (*P < 0.05). (TIF) [file ppat.1006022.s001.tif]

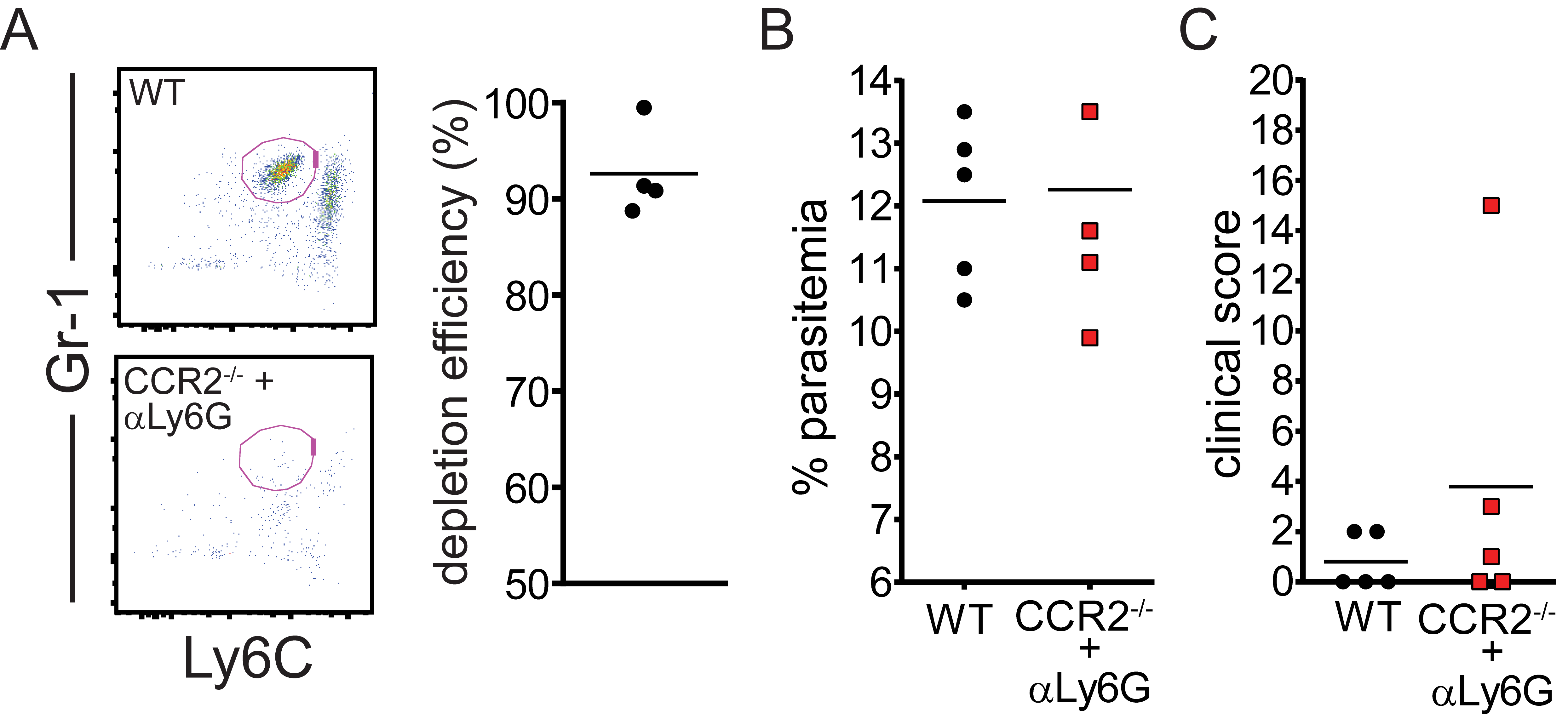

Supplement: S2 Fig — (A) Representative flow cytometric dot plots showing wild type (top) and neutrophil-depleted CCR2-deficient mice (bottom). Plots are gated on Thy1.2-CD11b+ cells. The right panel depicts a graphical representation of the depletion efficiency in the blood based on the following calculation: 100 –((%Thy1.2-Cd11b+Gr-1+Ly6C+ cells in the blood of each treated mouse divided by the average %Thy1.2-Cd11b+Gr-1+Ly6C+ cells in the blood of wild type mice) x 100). Blood parasitemia percentages (B) and clinical score (C) in wild type vs. neutrophil-depleted CCR2-/- mice at d6 p.i. (n = 5 mice per group; two independent experiments). (TIF) [file ppat.1006022.s002.tif]

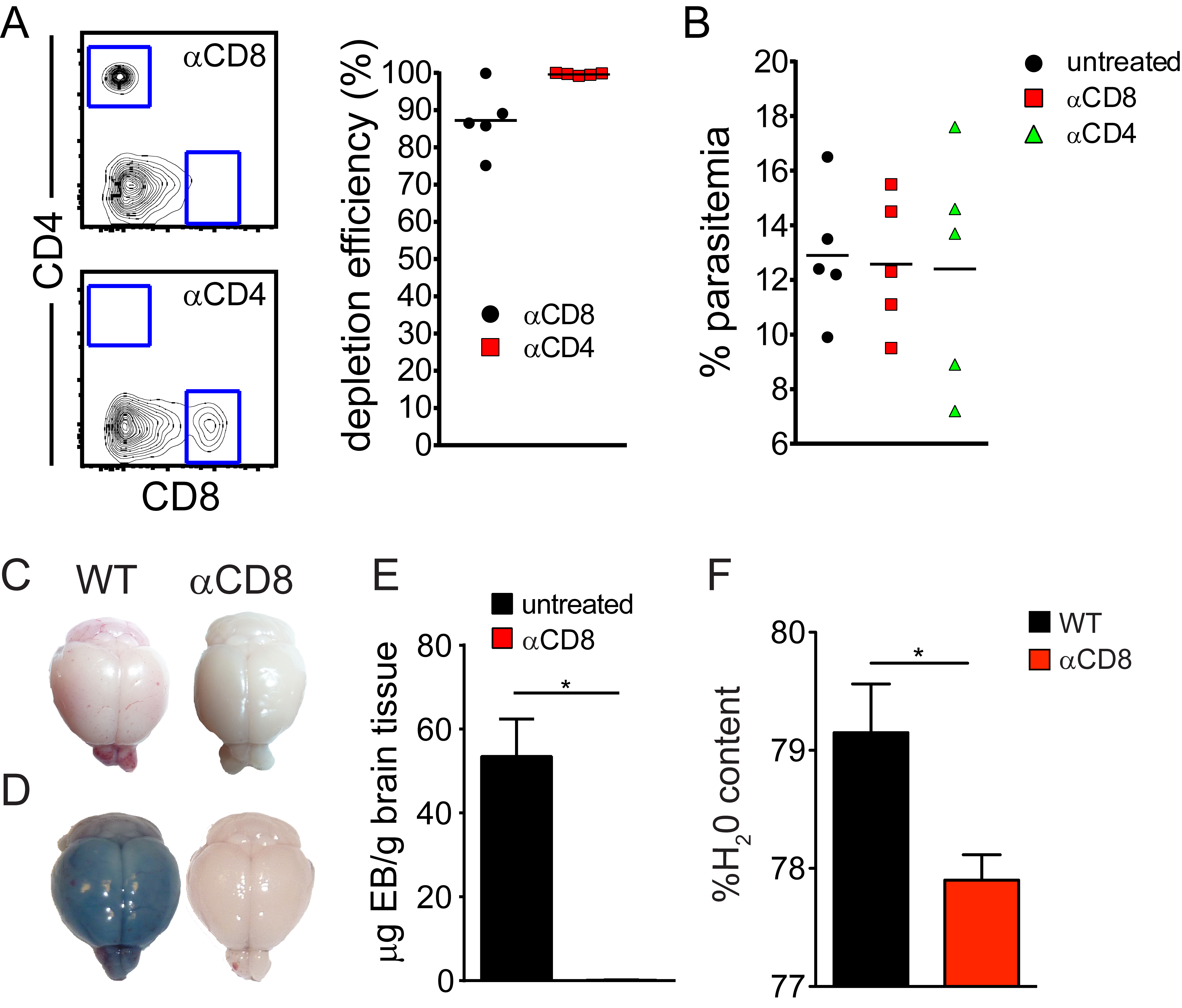

Supplement: S3 Fig — (A) Representative flow cytometric dot plots showing depletion of CD8+ (top) and CD4+ (bottom) T cells in the blood. The right panel is a graphical representation of the depletion efficiency. (B) Blood parasitemia was quantified in the following d6 p.i. mice described in Fig 3A: untreated B6, B6 + anti-CD4, B6 + anti-CD8. (n = 4–5 mice per group). (C) Representative brains from wild type (left) and CD8+ T cell depleted (right) mice at d6 p.i. (n = 5 mice per group). Note the absence of vascular hemorrhaging in CD8+ T cell depleted mice. (D) Representative brains from wild type (left) and CD8+ T cell depleted (right) mice at d6 p.i. following i.v. injection of Evans Blue dye. (E) Fluorometric quantification of data shown in (D) (mean ± SD; n = 5 mice per group). Note the absence of BBB breakdown in CD8+ T cell depleted mice. (F) Quantification of brain water content from wild type and CD8+ T cell depleted mice at d6 p.i. (mean ± SD; n = 5 mice per group). All data in this figure are representative of two independent experiments. (TIF) [file ppat.1006022.s003.tif]

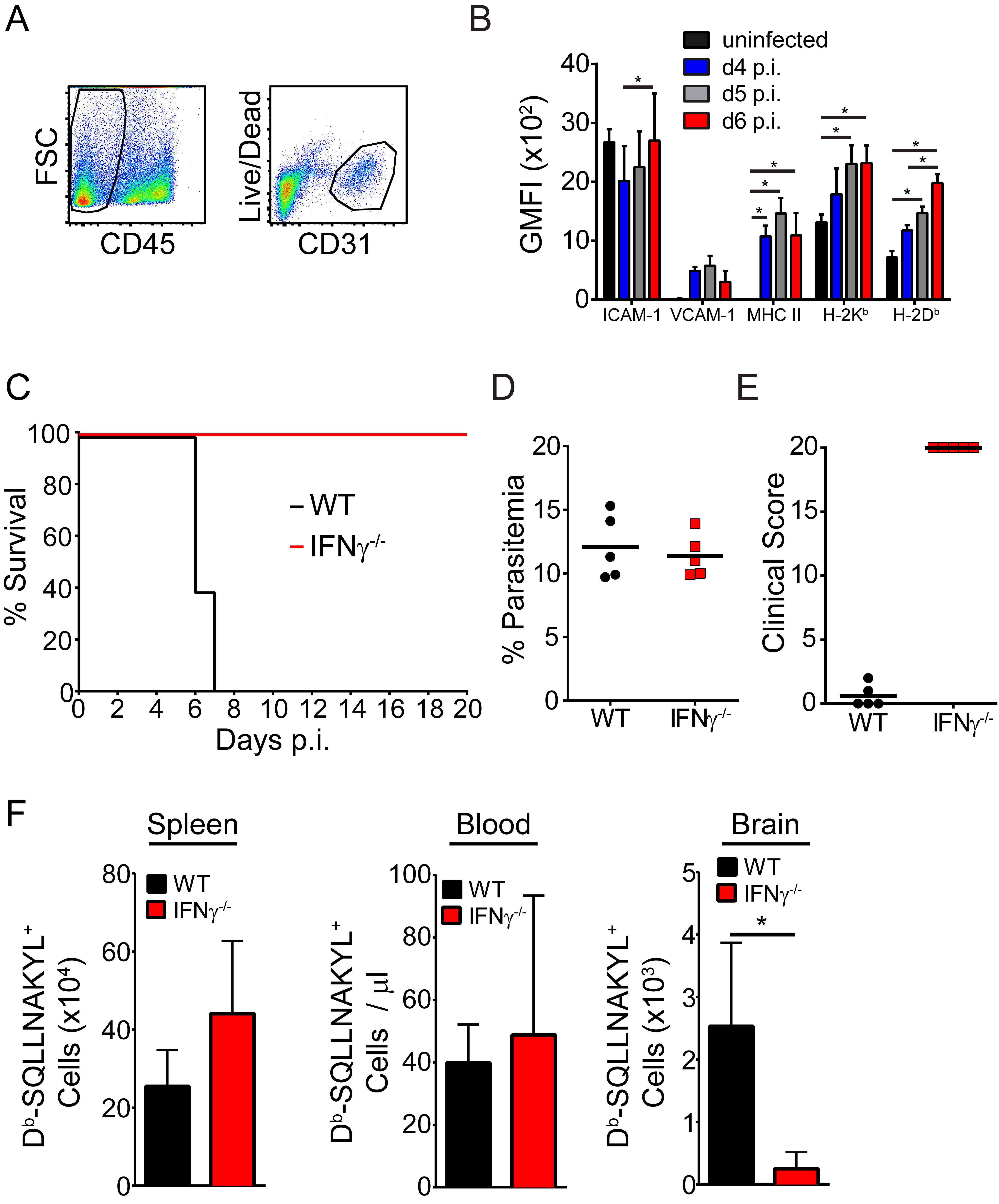

Supplement: S4 Fig — (A) Representative flow cytometric dot plots show the gating strategy used to identify ECs in the CNS. (B) The bar graph depicts the geometric mean fluorescent intensity of the denoted molecules on meningeal ECs extracted from naïve, d4 p.i., d5 p.i., and d6 p.i. B6 mice (mean ± SD; n = 3–4 mice per group). (C) Survival curve showing PbA-infected wild type and IFNγ-/- mice over time (n = 5 mice per group). (D and E) Blood parasitemia (D) and clinical scores (E) for the d6 p.i. wild type and IFNγ-/- mice shown in Fig 6D (n = 5 mice per group). (F) Graphical representation of Db-SQLLNAKYL+ CD8 T cells in the spleen, blood, and brain of WT and IFNγ mice at d6 p.i. All data in this figure are representative of two independent experiments. Asterisks denote statistical significance (*P < 0.05). (TIF) [file ppat.1006022.s004.tif]

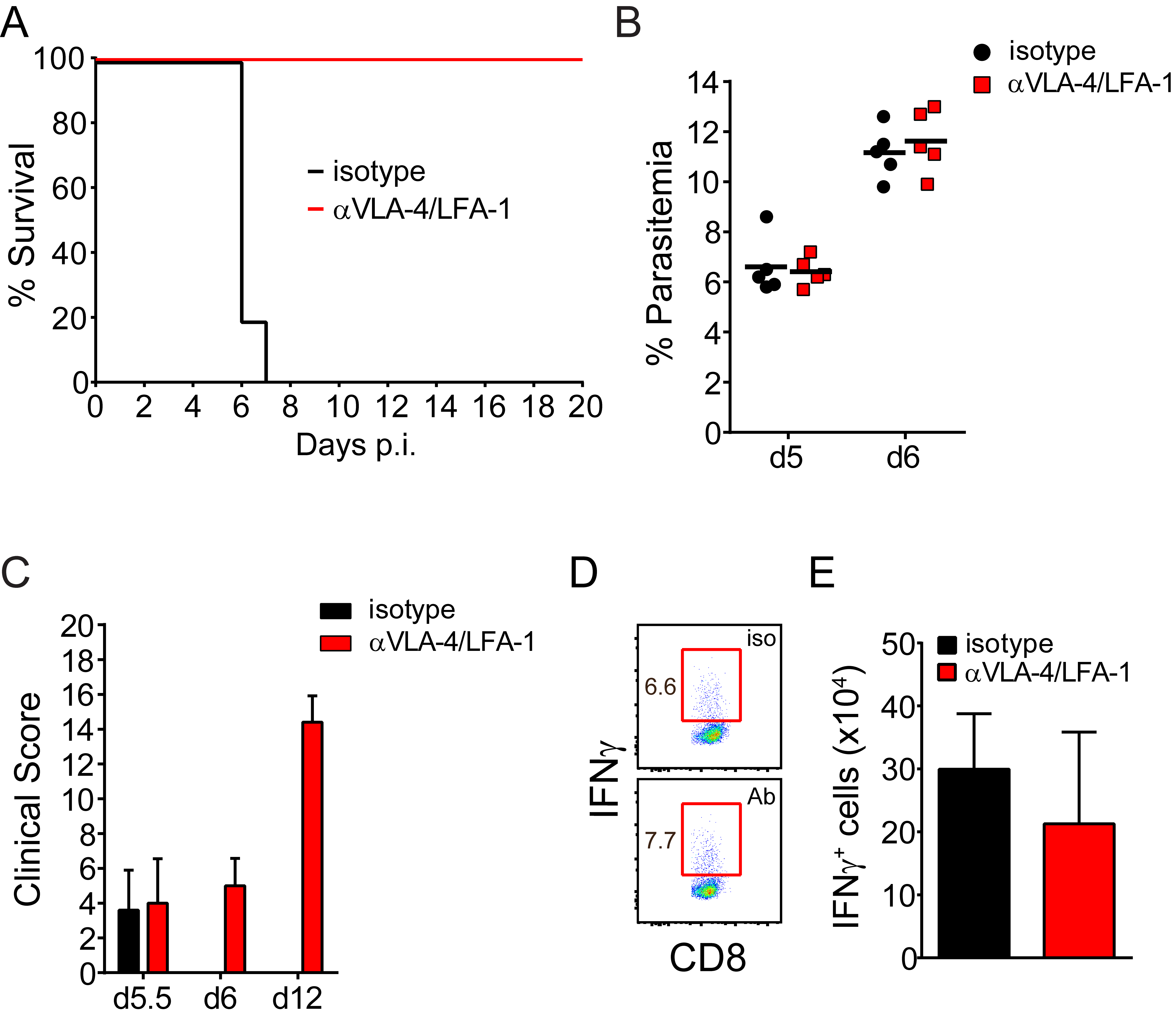

Supplement: S5 Fig — (A) Survival curve showing PbA-infected mice treated with 500 μg anti-VLA-4 + 500 μg anti-LFA-1 (red) or isotype control (black) antibodies i.v on day 5.5 and then again on day 6.5 p.i. Data are representative of three independent experiments with 5 mice per group. (B) Blood parasitemia levels in PbA-infected mice treated with 500 μg anti-VLA-4 + 500 μg anti-LFA-1 (red) or isotype control (black) antibodies i.v at day 5.5 and 6.5 p.i. (n = 5 mice per group; 3 independent experiments). (C) Clinical scores in the same mice assessed at d5.5, d6, and d12 p.i. (D) Representative dot plots of splenic CD8+ T cell IFNγ expression following in vitro stimulation with SQLLNAKYL peptide at d6 p.i. from mice treated with blocking or isotype control antibodies at d5.5 p.i. Plots are gated on live CD45+Thy1.2+CD8+ cells. (E) Quantification of data shown in (D) (mean ± SD; n = 5 mice per group). Data are representative of two independent experiments. (TIF) [file ppat.1006022.s005.tif]

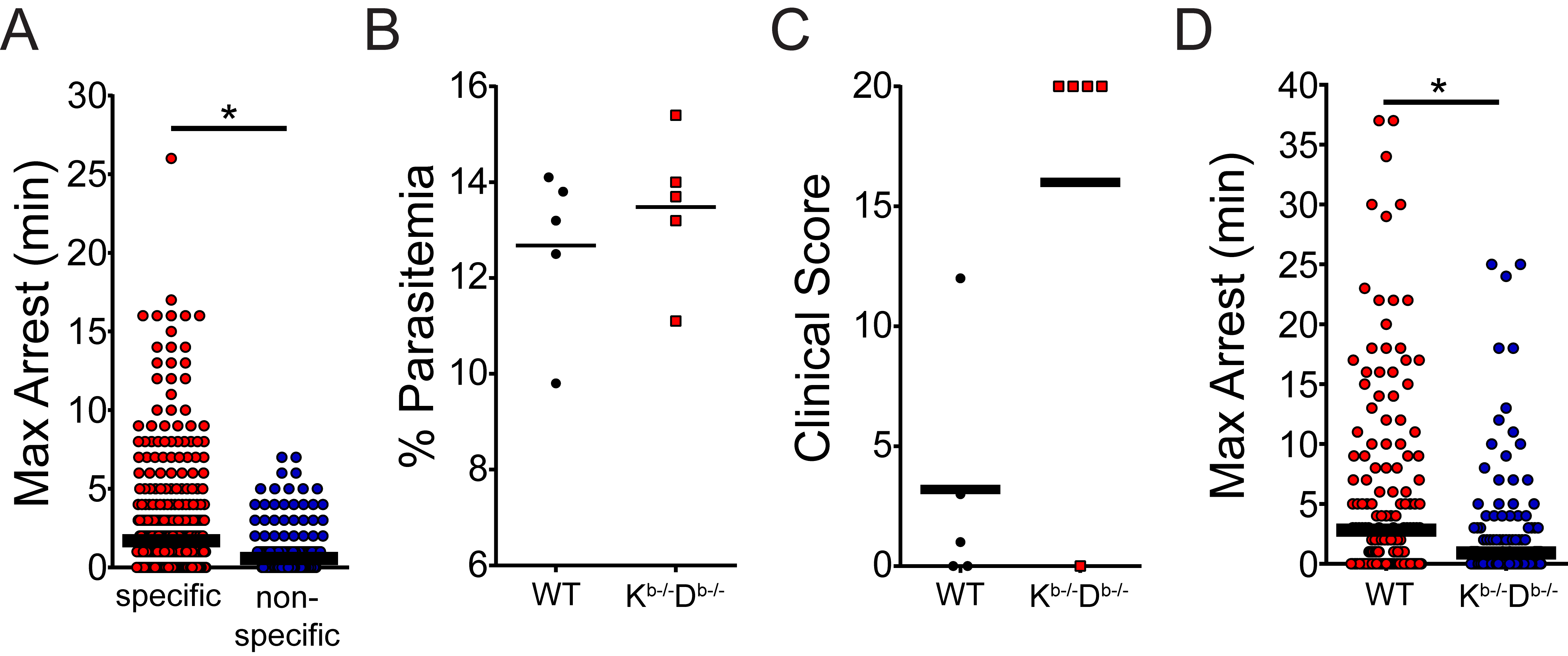

Supplement: S6 Fig — (A) Quantification of maximum arrest time for PbA-specific CD8 T cells within the cerebral vasculature of d6 p.i. mice before and after i.v. injection of anti-Kb-SIINFEKL or isotype control antibodies. Each dot represents an individual T cell, and horizontal black bars denote the group mean. Data are representative of 4 independent experiments. (B) Blood parasitemia and (C) clinical scores in PbA-infected WT→WT and WT→Kb-/-Db-/- BM chimeras at d6 p.i. (n = 5 per group; 2 independent experiments). See corresponding Fig 8F. (D) Quantification of maximum arrest time for PbA-specific CD8+ T cells within the cerebral vasculature of WT→WT and WT→Kb-/-Db-/- BM chimeras at d6 p.i. Each dot represents an individual T cell, and horizontal black bars denote the group mean. Data are representative of 7 independent experiments. Asterisks denote statistical significance (*P < 0.05). (TIF) [file ppat.1006022.s006.tif]
